# Supplementary material for: Stability Assessment of Four Chimeric Proteins for Human Chagas Disease Immunodiagnosis
Source: Biosensors (Basel). 2021 Aug 22;11(8):289. doi: 10.3390/bios11080289 (PMC8391164; doi:10.3390/bios11080289)
Supplement: Supplementary file 1 [file biosensors-11-00289-s001.zip › biosensors-1250906-supplementary.pdf]

Circular Dichroism (CD) Measurements

Wavelength (nm)	IBMP-8.1		IBMP-8.2		IBMP-8.3		IBMP-8.4	
	Preheating	Post-heating	Preheating	Post-heating	Preheating	Post-heating	Preheating	Post-heating
202	-27.97013	-25.97853	-91.5756	-78.2799	-36.6587	-3.38259	-26.5874	-26.9006
203	-24.54188	-23.24018	-96.4517	-101.886	-35.9421	-3.19263	-26.6327	-27.3876
204	-22.01187	-20.68987	-102.553	-99.6132	-34.631	-3.62961	-26.6751	-26.9014
205	-19.86683	-18.46263	-96.4023	-103.162	-33.4646	-4.17327	-25.9623	-25.8974
206	-16.6321	-16.1475	-94.9932	-97.0422	-30.1428	-4.28723	-25.0191	-25.0793
207	-15.04768	-15.51208	-91.8323	-90.83	-27.7089	-4.09431	-23.9768	-23.8166
208	-13.52575	-12.87615	-88.4071	-85.0746	-25.6183	-4.0586	-22.3295	-22.0939
209	-10.56511	-10.56681	-79.3097	-79.5211	-23.8265	-4.39545	-20.57	-19.8563
210	-9.305184	-9.869684	-75.5253	-74.4332	-21.5594	-4.47324	-18.9619	-18.0897
211	-7.976234	-8.078684	-69.2449	-66.3727	-19.2078	-4.05667	-17.5119	-16.7286
212	-7.151488	-6.692618	-64.0829	-62.5157	-17.1061	-4.03541	-16.1937	-15.4203
213	-5.790762	-6.148442	-62.3679	-57.7456	-15.7662	-4.60156	-15.3406	-14.3031
214	-4.837254	-5.608364	-60.6171	-56.0939	-14.8035	-4.35573	-14.7916	-13.8221
215	-4.382251	-4.559431	-58.3905	-55.8655	-13.7702	-4.27797	-14.2621	-13.3884
216	-3.639093	-3.749733	-57.0566	-54.4015	-12.7442	-4.40531	-13.8015	-13.0315
217	-3.483388	-3.581528	-56.3543	-53.95	-11.8938	-4.47391	-13.5486	-12.8941
218	-2.867548	-3.145068	-57.1802	-53.5615	-11.5213	-4.7132	-13.5479	-12.7812
219	-2.681349	-2.592629	-57.5655	-53.5026	-11.1679	-4.94468	-13.6792	-12.7993
220	-2.787281	-2.262941	-57.354	-53.2241	-10.6144	-4.9751	-13.6927	-12.7387
221	-2.579022	-1.891182	-57.3322	-52.7008	-10.4472	-5.00567	-13.3903	-12.5768
222	-2.632862	-2.245902	-57.085	-52.7603	-10.0647	-4.98418	-13.2062	-12.3171
223	-2.41908	-2.14955	-56.7443	-52.8708	-9.8156	-4.95346	-12.8953	-12.453

224	-2.351784	-2.173024	-55.5553	-51.7921	-9.53054	-4.78289	-12.6349	-12.018
225	-2.329289	-2.242849	-54.0074	-49.7915	-8.80413	-4.92819	-12.206	-11.4102
226	-2.093682	-2.239682	-51.6285	-47.4799	-8.3192	-4.68455	-11.5215	-10.7103
227	-2.320894	-2.083544	-48.7901	-45.0799	-7.74391	-4.44168	-10.7722	-10.0756
228	-2.119794	-2.036674	-45.6736	-42.1849	-7.19263	-3.96891	-9.99317	-9.10003
229	-1.878951	-1.986711	-41.8409	-38.7435	-6.58049	-3.85226	-8.97203	-8.26187
230	-1.686606	-1.856686	-37.8996	-34.9024	-5.72122	-3.67541	-7.95412	-7.32698
231	-1.439307	-1.623467	-33.6667	-31.0525	-4.68428	-3.21976	-6.95349	-6.48675
232	-1.493073	-1.508533	-29.8521	-27.425	-4.13042	-2.5174	-5.85922	-5.6788
233	-1.209538	-1.422368	-26.1767	-23.8071	-3.50015	-2.24303	-4.74324	-4.89359
234	-1.05638	-1.36933	-22.3053	-20.4232	-2.89223	-1.90696	-3.99586	-4.17189
235	-0.997638	-1.075638	-18.8895	-17.2385	-2.42992	-1.38961	-3.37402	-3.26231
236	-0.977393	-1.031733	-15.9468	-14.2302	-2.13414	-1.07402	-2.65314	-2.50525
237	-0.625726	-0.833646	-13.0026	-11.7438	-1.75557	-0.88587	-1.95443	-2.01386
238	-0.528288	-0.641218	-10.4322	-9.63963	-1.35974	-0.80377	-1.56866	-1.58942
239	-0.64198	-0.62988	-8.56131	-7.76363	-1.08464	-0.61211	-1.22489	-1.18204
240	-0.55298	-0.65311	-6.98277	-6.13522	-0.71008	-0.43066	-0.83796	-0.93186
241	-0.355128	-0.571419	-5.47755	-4.7611	-0.34507	-0.31294	-0.62773	-0.7473
242	-0.085212	-0.295631	-4.16836	-3.65132	-0.18711	-0.10174	-0.47856	-0.53066
243	-0.143813	-0.205337	-3.15634	-2.63347	-0.10394	0.043687	-0.23193	-0.37215
244	-0.048221	-0.087314	-2.47054	-2.17503	0.099468	0.126217	-0.14772	-0.31409
245	0.043654	-0.134019	-1.54143	-1.72224	0.060194	0.078276	-0.00566	-0.18929
246	-0.043568	-0.182423	-1.06941	-0.99877	-0.0476	0.196376	0.137935	-0.11391
247	-0.054866	-0.145671	-0.61105	-0.55526	0.132619	0.287938	0.297124	0.060513
248	0.037066	-0.149854	-0.33784	-0.3117	0.284396	0.321521	0.212932	0.018733
249	0.251396	-0.028694	-0.15049	-0.0019	0.330269	0.04026	0.176231	-0.0183
250	0.222026	-0.071003	0.244411	0.098598	0.415874	-0.0824	0.141399	0.159519

251	0.124874	0.023677	0.23936	0.086574	0.466899	0.135677	0.251583	0.18906
252	0.031567	0.139584	0.22813	0.311596	0.579922	0.259457	0.354977	0.260036
253	0.274036	0.211353	0.344608	0.296131	0.487461	0.235889	0.415493	0.198192
254	0.292236	0.175582	0.337005	0.469726	0.453903	0.215728	0.392654	0.208671
255	0.158212	0.143503	0.378626	0.58082	0.470621	0.450723	0.433244	0.22455
256	0.289228	0.240293	0.599439	0.547773	0.364759	0.337552	0.492635	0.218725
257	0.245775	0.175798	0.555253	0.591417	0.431581	0.224992	0.606157	0.228448
258	0.138204	0.192342	0.526571	0.522024	0.29981	0.421667	0.460394	0.209699
259	0.144018	0.274618	0.593176	0.475088	0.567556	0.33959	0.452093	0.290418
260	0.263287	0.411648	0.754437	0.766827	0.698849	0.308666	0.536869	0.267544

Dynamic Light Scattering (DLS)

Radius (nm)	IBMP-8.1		IBMP-8.2		IBMP-8.3		IBMP-8.4	
	Preheating	Post-heating	Preheating	Post-heating	Preheating	Post-heating	Preheating	Post-heating
0.0260057	0	0	0	0	0	0	0	0
0.0329794	0	0	0	0	0	0	0	0
0.0418231	0	0	0	0	0	0	0	0
0.0530384	0	0	0	0	0	0	0	0
0.0672611	0	0	0	0	0	0	0	0
0.0852978	0	0	0	0	0	0	0	0
0.108171	0	0	0	0	0	0	0	0
0.137178	0	0	0	0	0	0	0	0
0.173964	0	0	0	0	0	0	0	0
0.220614	0	0	0	0	0	0	0	0
0.279774	0	0	0	0	0	0	0	0
0.354797	0	0	0	0	0	0	0	0
0.44994	0	0	0	0	0	0	0	0
0.570595	0	0	0	0	0	0	0	0
0.723605	0	0	0	0	0	0	0	0
0.917647	0	0	0	0	0	0	0	0
1.16372	0	0	0	0	0	0	0	0
1.47579	0	0	0	0	0	0	0	0
1.87153	5.4047	0	0	0	0	0	0	0
2.3734	83.0882	48.8947	0.405836	0	0	0	0	0
3.00985	30.4137	3.32702	44.7228	0	9.71305	0	21.3563	24.5768
3.81697	9.25165	0	35.212	0	55.3663	42.9181	34.0492	32.0724

4.84052	1.49334	0	15.2969	0	27.5309	35.6311	22.6861	20.6111
6.13855	0	0	3.89654	0	0	0	10.1386	9.21134
7.78466	0	0	0.251547	0	0	0	3.27583	3.02907
9.87219	0	0	0	0	0	0	0.677972	0.655435
12.5195	0	0	0	4.73424	0	0	0.0318219	0.0429102
15.8767	0	0	0	0	0	0	0	0
20.1342	0	0	0	0	0	0	0	0
25.5334	0	0	0	0	0	0	0	0
32.3804	0	0	0	0	0	0	0	0
41.0635	0	0	0	0	0	0	0	0
52.0751	0	0	0	0	0	0	0	0
66.0395	0	0	0	0	0	0	0	0
83.7486	0	0	0	0	0	0	0	0
106.207	0	0	0	0	0	0	0	0
134.687	0	0	0	0	0	0	0	0
170.804	0	0	0	0	0	0	0	0
216.607	0	0	0	0	0	0	0	0
274.692	0	0	0	0	0	0	0	0
348.354	0	0	0	0	0	0	0	0
441.768	0	0	0	0	0	0	0	0
560.232	0	0	0	0	0	0	0	0
710.463	0	0	0	0	0	0	0	0
900.981	0	0	0	0	0	0	0	0
1142.59	0	0	0	0	0	0	0	0
1448.98	0	0	0	0	0.284396	0.321521	0	0
1837.54	0	0	0	0	0.330269	0.04026	0	0
2330.29	0	0	0	0	0.415874	-0.0824	0	0

2955.18	0	0	0	0	0.466899	0.135677	0	0
3747.64	0	0	0	0	0.579922	0.259457	0	0
4752.61	0	0	0.00135379	0	0.487461	0.235889	0	0
6027.06	0	0	0.0100637	0	0.453903	0.215728	0	0
7643.28	0	0	0.0433948	0	0.470621	0.450723	0	0
9692.89	0	0	0.0363213	0	0.364759	0.337552	0	0
12292.1	0	0	0	0	0.431581	0.224992	0	0
15588.4	0	0.192342	0.526571	0.522024	0.29981	0.421667	0	0
19768.5	0	0.274618	0.593176	0.475088	0.567556	0.33959	0	0

Indirect ELISA

Samples	IBMP-8.1				IBMP-8.2				IBMP-8.3				IBMP-8.4			
	Time of exposure (min)				Time of exposure (min)				Time of exposure (min)				Time of exposure (min)			
	0	1	10	20	0	1	10	20	0	1	10	20	0	1	10	20
Trypanosoma cruzi-negative 01	0.42	0.43	0.44	0.26	0.27	0.43	0.34	0.36	0.57	0.29	0.53	0.22	0.56	0.44	0.44	0.41
Trypanosoma cruzi-negative 02	0.27	0.27	0.08	0.19	0.21	0.31	0.19	0.17	0.21	0.4	0.15	0.07	0.18	0.14	0.26	0.15
Trypanosoma cruzi-negative 03	0.35	0.39	0.5	0.61	0.26	0.28	0.34	0.18	0.36	0.44	0.22	0.1	0.75	0.78	0.37	0.28
Trypanosoma cruzi-negative 04	0.23	0.22	0.1	0.21	0.7	0.26	0.56	0.33	0.2	0.21	0.09	0.05	0.2	0.12	0.2	0.14
Trypanosoma cruzi-negative 05	0.23	0.28	0.16	0.2	0.46	0.31	0.19	0.39	0.2	0.26	0.31	0.56	0.85	0.99	0.24	0.13
Trypanosoma cruzi-negative 06	0.39	0.3	0.26	0.33	0.23	0.42	0.24	0.22	0.26	0.24	0.23	0.12	0.3	0.21	0.25	0.34
Trypanosoma cruzi-negative 07	0.25	0.44	0.46	0.43	0.23	0.3	0.26	0.26	0.2	0.21	0.12	0.07	0.27	0.17	0.24	0.16
Trypanosoma cruzi-negative 08	0.25	0.31	0.27	0.24	0.29	0.31	0.23	0.25	0.19	0.22	0.31	0.15	0.27	0.23	0.22	0.15
Trypanosoma cruzi-negative 09	0.32	0.33	0.52	0.3	0.33	0.32	0.86	0.48	0.23	0.32	0.16	0.09	0.22	0.19	0.28	0.17
Trypanosoma cruzi-negative 10	0.46	0.45	0.34	0.32	0.26	0.3	0.35	0.24	0.2	0.27	0.22	0.1	0.3	0.31	0.36	0.43
Trypanosoma cruzi-negative 11	0.28	0.34	0.46	0.38	0.33	0.41	0.28	0.24	0.24	0.29	0.23	0.12	0.2	0.41	0.5	0.29
Trypanosoma cruzi-negative 12	0.26	0.26	0.44	0.47	0.38	0.4	0.24	0.22	0.22	0.23	0.19	0.08	0.2	0.16	0.27	0.21
Trypanosoma cruzi-negative 13	0.52	0.59	0.45	0.53	0.26	0.35	0.44	0.31	0.43	0.33	0.29	0.14	0.33	0.3	0.44	0.32
Trypanosoma cruzi-negative 14	0.24	0.26	0.45	0.28	0.45	0.35	0.18	0.48	0.2	0.3	0.11	0.06	0.16	0.15	0.23	0.15
Trypanosoma cruzi-negative 15	0.27	0.27	0.49	0.43	0.25	0.36	0.88	0.18	0.22	0.21	0.15	0.08	0.21	0.15	0.21	0.17
Trypanosoma cruzi-negative 16	0.26	0.26	0.11	0.33	0.5	0.36	0.21	0.59	0.23	0.29	0.19	0.1	0.18	0.17	0.25	0.18
Trypanosoma cruzi-negative 17	0.27	0.3	0.11	0.24	0.3	0.32	0.22	0.32	0.21	0.25	0.23	0.11	0.28	0.25	0.24	0.24
Trypanosoma cruzi-negative 18	0.23	0.31	0.46	0.24	0.25	0.28	0.82	0.27	0.2	0.4	0.13	0.07	0.21	0.17	0.2	0.14
Trypanosoma cruzi-negative 19	0.17	0.16	0.31	0.17	0.42	0.39	0.81	0.34	0.16	0.19	0.07	0.06	0.18	0.18	0.16	0.13
Trypanosoma cruzi-negative 20	0.3	0.3	0.45	0.4	0.24	0.43	0.18	0.29	0.46	0.28	0.1	0.08	0.18	0.18	0.2	0.22

Trypanosoma cruzi-negative 21	0.31	0.34	0.52	0.54	0.44	0.38	0.23	0.48	0.24	0.38	0.17	0.1	0.22	0.24	0.29	0.23
Trypanosoma cruzi-negative 22	0.24	0.28	0.45	0.21	0.19	0.32	0.79	0.2	0.19	0.23	0.08	0.07	0.21	0.22	0.16	0.13
Trypanosoma cruzi-positive 01	1.24	1.13	0.62	0.62	5.13	5.85	9.03	5.99	1.23	1.15	0.89	0.36	1.05	0.83	0.92	0.89
Trypanosoma cruzi-positive 02	4.44	4.29	3.52	2.25	3.37	3.94	5.24	4.43	3.93	4.11	4.69	3.4	3.61	2.99	7.5	4.52
Trypanosoma cruzi-positive 03	5.54	5.63	4.73	2.18	2.35	3.27	2.6	2.9	3.82	4.48	1.08	3.14	3.42	3	6.76	5.64
Trypanosoma cruzi-positive 04	3.61	3.6	2.63	1.75	1.69	3.01	1.44	1.67	2.95	3.4	2.07	2.53	2.87	2.66	3.87	3.92
Trypanosoma cruzi-positive 05	2.76	2.64	1.86	1.25	3.67	4.26	4.54	3.16	3.04	3.66	2.03	2.82	3.26	2.69	4.38	4.38
Trypanosoma cruzi-positive 06	3.25	3.17	0.76	1.01	3.24	4.25	4.89	4.03	3.08	3.62	5.48	2.33	2.91	2.67	4.74	4.36
Trypanosoma cruzi-positive 07	3.36	2.92	1.94	1.46	2.72	3.25	3.38	3.7	3.08	3.31	6.8	3.17	3.41	2.94	4.54	4.91
Trypanosoma cruzi-positive 08	3.31	3.55	3.83	1.24	2.15	2.47	1.98	2.24	3.34	4.05	2.25	3.09	3.3	3	5.77	4.72
Trypanosoma cruzi-positive 09	2.85	2.56	1.47	3.05	4.65	5.66	7.41	6.51	2.46	2.69	6.91	3.19	3.29	2.83	2.9	3.04
Trypanosoma cruzi-positive 10	3.32	3.09	1.77	2.97	3.62	4.4	4.62	3.72	2.7	3.16	6.55	2.95	3.04	2.73	3.77	3.34
Trypanosoma cruzi-positive 11	2.96	3.27	1.49	1.18	2.32	2.75	2.76	3.38	2.49	2.95	2.95	1.23	2	1.69	3.45	2.57
Trypanosoma cruzi-positive 12	2.77	2.88	1.57	2.69	1.49	2	1.42	1.39	2.51	2.99	5.35	2.36	2.84	2.59	3.04	2.85
Trypanosoma cruzi-positive 13	1.61	1.44	0.91	0.92	4.78	5.53	7.46	6.61	2.07	2.39	5.79	2.7	2.98	2.73	2.36	2.34
Trypanosoma cruzi-positive 14	3.55	3.58	2.02	2.82	2.49	3.15	3.36	3.57	2.96	3.52	5.66	2.37	2.94	2.69	4.18	3.87
Trypanosoma cruzi-positive 15	3.55	3.86	2.74	1.73	3.58	4.97	5.56	4.49	2.92	3.44	1.37	3.54	3.49	3.14	4.35	3.33
Trypanosoma cruzi-positive 16	5.13	5.55	4.97	2.29	3.45	5.11	4.88	2.99	4.17	5.11	2.45	2.89	3.23	2.87	8.37	7.16
Trypanosoma cruzi-positive 17	2.27	2.39	1.21	2.24	2.63	3.11	2.92	2.41	1.93	2.09	3.54	1.7	2.33	2.13	1.87	2
Trypanosoma cruzi-positive 18	3.1	2.73	1	1.92	4.68	5.68	8.24	6.1	2.78	3.44	2	3.34	3.32	3.08	4	3.96
Trypanosoma cruzi-positive 19	2.76	2.99	1.49	0.83	1	1.04	0.74	0.67	2.47	3.22	2.12	2.88	2.99	2.79	3.34	3.37
Trypanosoma cruzi-positive 20	1.57	2.25	0.65	1.22	3.35	4.15	5.02	4.69	2	3.44	2.49	1.2	1.87	2	2.35	2.33
Trypanosoma cruzi-positive 21	2.83	2.93	0.99	2.69	1.8	2.28	1.83	1.88	2.7	2.99	5.31	2.77	2.93	2.82	2.65	2.94
Trypanosoma cruzi-positive 22	2.43	2.52	0.83	2.32	2.24	2.8	2.84	2.76	3.15	3.95	2.34	2.97	3.37	3.17	4.73	4.14

Circular Dichroism (CD) Measurements

Wavelength (nm)	IBMP-8.1				IBMP-8.2				IBMP-8.3				IBMP-8.4			
	Time of exposure (hours)				Time of exposure (hours)				Time of exposure (hours)				Time of exposure (hours)			
	**0**	**24**	**48**	**72**	**0**	**24**	**48**	**72**	**0**	**24**	**48**	**72**	**0**	**24**	**48**	**72**
193	-21.981	-21.790	-19.844	-18.598	No data	No data	No data	No data	-31.343	-29.538	-27.606	-32.730	0.005	-3.955	-1.244	-3.994
193,5	-22.512	-21.841	-20.540	-18.962	No data	No data	No data	No data	-32.533	-31.931	-29.096	-33.158	-1.575	-4.959	-2.622	-4.842
194	-22.997	-22.018	-21.379	-19.336	No data	No data	No data	No data	-34.603	-34.602	-31.164	-33.929	-3.428	-6.064	-4.275	-6.200
194,5	-23.624	-22.321	-22.281	-20.035	No data	No data	No data	No data	-37.004	-37.466	-33.475	-35.323	-5.727	-7.639	-6.406	-7.803
195	-24.114	-23.034	-22.986	-20.981	-5.280	-4.141	0.410	0.358	-39.055	-39.185	-35.288	-36.925	-7.745	-9.048	-8.490	-9.547
195,5	-24.545	-23.559	-23.501	-21.951	-10.888	-7.368	-3.148	-3.812	-41.296	-40.886	-37.589	-38.760	-9.914	-10.691	-9.641	-11.286
196	-25.085	-24.444	-23.903	-22.805	-17.173	-11.681	-7.671	-8.929	-44.066	-42.556	-40.091	-41.350	-12.006	-12.494	-11.406	-12.991
196,5	-25.745	-25.065	-24.244	-23.744	-21.756	-16.915	-13.475	-15.004	-46.268	-44.340	-42.283	-43.865	-14.129	-14.542	-13.250	-14.448
197	-26.148	-25.547	-24.583	-24.326	-25.369	-22.146	-18.844	-20.524	-48.172	-45.999	-44.777	-45.663	-16.101	-16.322	-15.089	-15.893
197,5	-26.393	-25.701	-24.904	-24.677	-28.458	-27.212	-24.281	-25.257	-49.576	-47.673	-46.803	-46.990	-17.748	-17.713	-16.735	-17.151
198	-26.332	-25.817	-25.103	-24.663	-31.530	-31.761	-29.337	-29.472	-51.231	-48.994	-48.708	-48.023	-19.265	-18.858	-18.074	-18.323
198,5	-26.120	-25.863	-25.096	-24.535	-35.121	-35.790	-33.958	-33.330	-52.488	-49.978	-50.108	-48.574	-20.504	-19.918	-19.175	-19.265
199	-25.625	-25.840	-24.882	-24.261	-38.292	-39.345	-37.441	-36.623	-53.437	-50.719	-51.110	-49.029	-21.520	-20.814	-20.228	-20.063
199,5	-25.069	-25.639	-24.480	-23.916	-41.469	-42.328	-39.959	-39.629	-54.191	-51.419	-51.467	-49.556	-22.335	-21.661	-21.133	-20.801
200	-24.281	-25.128	-23.918	-23.508	-44.184	-44.729	-41.764	-41.924	-54.428	-51.877	-51.285	-49.807	-22.965	-22.371	-21.840	-21.427
200,5	-23.444	-24.321	-23.275	-23.107	-46.446	-46.627	-43.316	-43.775	-54.086	-52.130	-50.789	-49.878	-23.475	-22.925	-22.294	-21.935
201	-22.493	-23.307	-22.580	-22.527	-48.097	-48.118	-44.676	-45.131	-53.376	-52.002	-50.084	-49.658	-23.743	-23.255	-22.473	-22.220
201,5	-21.736	-22.341	-21.873	-21.762	-49.419	-49.257	-46.141	-46.303	-52.493	-51.557	-49.519	-49.207	-23.932	-23.347	-22.569	-22.346
202	-21.018	-21.517	-21.153	-20.885	-50.422	-50.068	-47.622	-47.333	-51.569	-50.839	-48.971	-48.527	-23.947	-23.263	-22.479	-22.252
202,5	-20.399	-20.742	-20.429	-19.977	-51.324	-50.549	-48.683	-48.188	-50.634	-49.867	-48.546	-47.899	-23.879	-23.048	-22.345	-22.016
203	-19.603	-19.958	-19.685	-19.093	-51.864	-50.713	-49.305	-48.630	-49.488	-48.624	-47.828	-46.866	-23.608	-22.778	-22.117	-21.622

203,5	-18.840	-19.168	-18.927	-18.287	-52.009	-50.668	-49.511	-48.878	-48.379	-47.320	-46.948	-45.686	-23.251	-22.473	-21.871	-21.196
204	-17.937	-18.396	-18.120	-17.563	-51.878	-50.458	-49.471	-48.759	-47.063	-45.977	-45.791	-44.164	-22.851	-22.096	-21.550	-20.787
204,5	-17.049	-17.481	-17.271	-16.860	-51.531	-50.148	-49.190	-48.430	-45.604	-44.603	-44.414	-42.599	-22.400	-21.633	-21.205	-20.376
205	-16.119	-16.524	-16.373	-16.058	-51.004	-49.720	-48.879	-47.849	-44.002	-43.217	-42.933	-40.905	-21.891	-21.121	-20.726	-19.956
205,5	-15.242	-15.554	-15.450	-15.078	-50.281	-49.160	-48.374	-47.053	-42.324	-41.765	-41.416	-39.214	-21.344	-20.549	-20.166	-19.471
206	-14.294	-14.565	-14.514	-14.023	-49.434	-48.406	-47.744	-46.071	-40.448	-40.163	-39.836	-37.508	-20.733	-19.939	-19.509	-18.919
206,5	-13.355	-13.548	-13.597	-12.941	-48.378	-47.459	-46.826	-44.934	-38.583	-38.441	-38.113	-35.850	-19.979	-19.308	-18.780	-18.217
207	-12.399	-12.557	-12.711	-11.929	-47.132	-46.324	-45.722	-43.629	-36.725	-36.602	-36.253	-34.159	-19.231	-18.632	-17.997	-17.396
207,5	-11.521	-11.652	-11.865	-11.023	-45.781	-45.066	-44.425	-42.276	-34.932	-34.820	-34.305	-32.471	-18.466	-17.918	-17.244	-16.551
208	-10.712	-10.879	-11.064	-10.280	-44.379	-43.727	-42.989	-40.894	-33.219	-33.030	-32.425	-30.918	-17.738	-17.178	-16.506	-15.772
208,5	-9.965	-10.202	-10.299	-9.657	-42.942	-42.342	-41.415	-39.507	-31.508	-31.309	-30.623	-29.455	-16.995	-16.372	-15.789	-15.041
209	-9.253	-9.555	-9.576	-9.145	-41.478	-40.931	-39.851	-38.117	-29.836	-29.604	-28.958	-28.076	-16.269	-15.529	-15.062	-14.374
209,5	-8.551	-8.919	-8.892	-8.634	-40.070	-39.506	-38.331	-36.729	-28.219	-28.011	-27.419	-26.689	-15.537	-14.683	-14.370	-13.720
210	-7.934	-8.259	-8.276	-8.095	-38.714	-38.087	-36.951	-35.335	-26.687	-26.430	-25.976	-25.259	-14.839	-13.908	-13.694	-13.096
210,5	-7.412	-7.591	-7.730	-7.513	-37.377	-36.717	-35.704	-33.992	-25.205	-24.973	-24.546	-23.833	-14.142	-13.243	-13.048	-12.484
211	-6.963	-6.988	-7.265	-6.909	-36.103	-35.434	-34.554	-32.759	-23.799	-23.556	-23.165	-22.419	-13.489	-12.703	-12.454	-11.932
211,5	-6.525	-6.465	-6.853	-6.330	-34.914	-34.273	-33.496	-31.697	-22.488	-22.232	-21.859	-21.092	-12.909	-12.263	-11.941	-11.435
212	-6.125	-6.034	-6.482	-5.803	-33.820	-33.256	-32.511	-30.763	-21.213	-20.978	-20.662	-19.900	-12.370	-11.883	-11.496	-11.015
212,5	-5.708	-5.677	-6.124	-5.387	-32.810	-32.388	-31.579	-29.963	-20.003	-19.873	-19.558	-18.846	-11.885	-11.512	-11.117	-10.635
213	-5.303	-5.372	-5.767	-5.046	-31.976	-31.650	-30.829	-29.301	-18.865	-18.864	-18.532	-17.921	-11.464	-11.137	-10.825	-10.326
213,5	-4.954	-5.062	-5.417	-4.776	-31.273	-31.025	-30.244	-28.775	-17.872	-17.936	-17.569	-17.061	-11.106	-10.761	-10.562	-10.039
214	-4.605	-4.724	-5.084	-4.458	-30.759	-30.495	-29.750	-28.335	-16.979	-17.064	-16.658	-16.216	-10.769	-10.408	-10.290	-9.739
214,5	-4.306	-4.418	-4.773	-4.162	-30.382	-30.046	-29.343	-28.011	-16.235	-16.271	-15.858	-15.451	-10.507	-10.130	-10.018	-9.451
215	-4.021	-4.141	-4.491	-3.851	-30.147	-29.666	-28.974	-27.736	-15.559	-15.535	-15.185	-14.757	-10.272	-9.910	-9.772	-9.211
215,5	-3.776	-3.912	-4.238	-3.589	-29.978	-29.353	-28.643	-27.517	-14.965	-14.851	-14.640	-14.123	-10.095	-9.740	-9.571	-8.981
216	-3.542	-3.709	-4.004	-3.361	-29.843	-29.112	-28.389	-27.263	-14.391	-14.199	-14.154	-13.538	-9.992	-9.576	-9.397	-8.803
216,5	-3.368	-3.554	-3.782	-3.192	-29.716	-28.934	-28.252	-27.060	-13.816	-13.589	-13.735	-13.020	-9.935	-9.445	-9.260	-8.656

217	-3.210	-3.364	-3.572	-3.022	-29.609	-28.819	-28.194	-26.884	-13.280	-13.050	-13.314	-12.524	-9.847	-9.320	-9.166	-8.562
217,5	-3.072	-3.175	-3.373	-2.835	-29.536	-28.749	-28.203	-26.784	-12.774	-12.546	-12.887	-12.056	-9.754	-9.209	-9.084	-8.480
218	-2.942	-2.968	-3.186	-2.664	-29.491	-28.707	-28.200	-26.694	-12.330	-12.109	-12.440	-11.593	-9.660	-9.086	-8.988	-8.433
218,5	-2.828	-2.781	-3.011	-2.477	-29.461	-28.671	-28.182	-26.653	-11.927	-11.750	-12.041	-11.193	-9.564	-8.989	-8.890	-8.358
219	-2.731	-2.612	-2.861	-2.319	-29.409	-28.637	-28.130	-26.627	-11.584	-11.447	-11.670	-10.812	-9.486	-8.904	-8.789	-8.265
219,5	-2.650	-2.467	-2.744	-2.186	-29.335	-28.593	-28.078	-26.632	-11.272	-11.159	-11.370	-10.486	-9.441	-8.851	-8.675	-8.177
220	-2.574	-2.364	-2.663	-2.108	-29.228	-28.553	-28.060	-26.654	-10.991	-10.904	-11.098	-10.217	-9.410	-8.816	-8.582	-8.099
220,5	-2.494	-2.292	-2.620	-2.071	-29.163	-28.519	-28.077	-26.687	-10.742	-10.671	-10.885	-10.045	-9.351	-8.791	-8.497	-8.038
221	-2.412	-2.266	-2.612	-2.068	-29.140	-28.492	-28.112	-26.720	-10.535	-10.485	-10.680	-9.907	-9.284	-8.786	-8.477	-8.046
221,5	-2.352	-2.277	-2.626	-2.065	-29.152	-28.455	-28.155	-26.726	-10.385	-10.338	-10.484	-9.826	-9.208	-8.771	-8.486	-8.095
222	-2.316	-2.358	-2.644	-2.073	-29.182	-28.396	-28.153	-26.673	-10.239	-10.205	-10.254	-9.720	-9.141	-8.722	-8.530	-8.159
222,5	-2.302	-2.432	-2.658	-2.043	-29.166	-28.288	-28.082	-26.518	-10.112	-10.055	-10.024	-9.566	-9.081	-8.641	-8.528	-8.176
223	-2.285	-2.479	-2.654	-1.989	-29.047	-28.116	-27.922	-26.309	-9.973	-9.885	-9.801	-9.332	-9.015	-8.544	-8.491	-8.135
223,5	-2.279	-2.475	-2.636	-1.924	-28.841	-27.868	-27.660	-26.029	-9.832	-9.666	-9.605	-9.072	-8.937	-8.447	-8.374	-8.018
224	-2.259	-2.429	-2.609	-1.893	-28.548	-27.548	-27.302	-25.712	-9.645	-9.443	-9.443	-8.827	-8.844	-8.355	-8.240	-7.865
224,5	-2.239	-2.372	-2.584	-1.895	-28.196	-27.170	-26.895	-25.363	-9.430	-9.224	-9.319	-8.629	-8.755	-8.278	-8.094	-7.673
225	-2.211	-2.315	-2.559	-1.946	-27.789	-26.738	-26.435	-24.987	-9.198	-9.039	-9.209	-8.459	-8.638	-8.189	-7.962	-7.489
225,5	-2.198	-2.286	-2.543	-1.969	-27.327	-26.262	-25.918	-24.570	-8.954	-8.857	-9.064	-8.298	-8.516	-8.091	-7.830	-7.307
226	-2.154	-2.262	-2.522	-1.967	-26.771	-25.740	-25.354	-24.099	-8.685	-8.674	-8.860	-8.120	-8.347	-7.914	-7.678	-7.100
226,5	-2.111	-2.249	-2.497	-1.907	-26.119	-25.166	-24.718	-23.531	-8.395	-8.446	-8.584	-7.893	-8.140	-7.678	-7.514	-6.898
227	-2.033	-2.204	-2.459	-1.822	-25.363	-24.529	-24.023	-22.862	-8.094	-8.191	-8.262	-7.641	-7.881	-7.384	-7.303	-6.689
227,5	-1.984	-2.168	-2.417	-1.717	-24.549	-23.831	-23.289	-22.124	-7.812	-7.929	-7.930	-7.382	-7.610	-7.079	-7.073	-6.493
228	-1.954	-2.115	-2.371	-1.652	-23.693	-23.069	-22.532	-21.350	-7.544	-7.680	-7.613	-7.122	-7.336	-6.781	-6.822	-6.279
228,5	-1.940	-2.069	-2.331	-1.628	-22.817	-22.254	-21.749	-20.568	-7.284	-7.430	-7.328	-6.866	-7.088	-6.523	-6.576	-6.063
229	-1.915	-2.038	-2.292	-1.647	-21.911	-21.396	-20.934	-19.783	-7.025	-7.173	-7.071	-6.611	-6.845	-6.271	-6.318	-5.847
229,5	-1.907	-2.022	-2.249	-1.694	-20.977	-20.497	-20.076	-18.988	-6.775	-6.914	-6.833	-6.357	-6.589	-6.023	-6.057	-5.610
230	-1.890	-1.999	-2.202	-1.754	-20.018	-19.562	-19.166	-18.177	-6.487	-6.645	-6.576	-6.093	-6.279	-5.770	-5.776	-5.361

230,5	-1.870	-1.984	-2.151	-1.787	-19.016	-18.597	-18.211	-17.320	-6.182	-6.355	-6.310	-5.837	-5.939	-5.505	-5.492	-5.107
231	-1.837	-1.957	-2.093	-1.772	-18.009	-17.610	-17.222	-16.438	-5.871	-6.060	-6.036	-5.578	-5.581	-5.220	-5.202	-4.864
231,5	-1.788	-1.919	-2.030	-1.720	-16.988	-16.614	-16.225	-15.528	-5.568	-5.767	-5.740	-5.314	-5.229	-4.950	-4.904	-4.599
232	-1.697	-1.852	-1.965	-1.632	-15.972	-15.624	-15.232	-14.597	-5.267	-5.452	-5.416	-5.010	-4.884	-4.661	-4.607	-4.321
232,5	-1.578	-1.783	-1.898	-1.550	-14.941	-14.653	-14.270	-13.655	-4.962	-5.122	-5.088	-4.692	-4.570	-4.377	-4.321	-4.030
233	-1.453	-1.700	-1.825	-1.469	-13.937	-13.705	-13.351	-12.725	-4.654	-4.768	-4.760	-4.375	-4.280	-4.066	-4.044	-3.736
233,5	-1.364	-1.638	-1.747	-1.421	-12.952	-12.782	-12.469	-11.819	-4.354	-4.413	-4.442	-4.055	-3.996	-3.766	-3.777	-3.444
234	-1.294	-1.560	-1.665	-1.370	-12.023	-11.874	-11.617	-10.955	-4.059	-4.089	-4.146	-3.738	-3.719	-3.474	-3.512	-3.165
234,5	-1.246	-1.495	-1.581	-1.305	-11.122	-10.985	-10.766	-10.134	-3.779	-3.789	-3.864	-3.430	-3.447	-3.204	-3.230	-2.903
235	-1.195	-1.412	-1.496	-1.211	-10.267	-10.116	-9.926	-9.360	-3.517	-3.506	-3.589	-3.141	-3.180	-2.950	-2.948	-2.644
235,5	-1.132	-1.334	-1.409	-1.123	-9.453	-9.274	-9.103	-8.618	-3.263	-3.249	-3.337	-2.885	-2.917	-2.727	-2.681	-2.415
236	-1.046	-1.237	-1.323	-1.028	-8.661	-8.472	-8.314	-7.899	-3.017	-3.006	-3.081	-2.648	-2.659	-2.520	-2.435	-2.204
236,5	-0.945	-1.152	-1.243	-0.944	-7.898	-7.724	-7.573	-7.194	-2.787	-2.772	-2.829	-2.434	-2.409	-2.315	-2.205	-2.017
237	-0.873	-1.068	-1.167	-0.862	-7.192	-7.040	-6.900	-6.539	-2.566	-2.551	-2.599	-2.254	-2.191	-2.123	-2.022	-1.856
237,5	-0.826	-0.997	-1.094	-0.793	-6.528	-6.419	-6.279	-5.941	-2.354	-2.354	-2.392	-2.103	-2.003	-1.947	-1.865	-1.729
238	-0.806	-0.959	-1.025	-0.749	-5.915	-5.862	-5.700	-5.409	-2.160	-2.189	-2.216	-1.975	-1.854	-1.783	-1.741	-1.608
238,5	-0.789	-0.946	-0.959	-0.700	-5.335	-5.357	-5.169	-4.925	-1.982	-2.039	-2.041	-1.841	-1.721	-1.614	-1.617	-1.479
239	-0.775	-0.937	-0.892	-0.672	-4.816	-4.892	-4.697	-4.476	-1.823	-1.891	-1.894	-1.709	-1.616	-1.465	-1.496	-1.343
239,5	-0.745	-0.924	-0.822	-0.647	-4.343	-4.451	-4.256	-4.039	-1.657	-1.741	-1.758	-1.570	-1.509	-1.335	-1.368	-1.202
240	-0.693	-0.901	-0.752	-0.632	-3.925	-4.028	-3.835	-3.623	-1.517	-1.573	-1.637	-1.428	-1.398	-1.218	-1.247	-1.059
240,5	-0.620	-0.838	-0.684	-0.585	-3.519	-3.622	-3.431	-3.237	-1.377	-1.408	-1.494	-1.268	-1.277	-1.105	-1.118	-0.931
241	-0.529	-0.748	-0.623	-0.530	-3.145	-3.240	-3.044	-2.871	-1.251	-1.238	-1.346	-1.123	-1.156	-0.998	-1.001	-0.813
241,5	-0.443	-0.638	-0.567	-0.454	-2.774	-2.889	-2.685	-2.546	-1.115	-1.089	-1.184	-0.988	-1.018	-0.885	-0.901	-0.709
242	-0.357	-0.534	-0.518	-0.385	-2.430	-2.574	-2.362	-2.236	-0.985	-0.960	-1.016	-0.871	-0.881	-0.778	-0.814	-0.628
242,5	-0.290	-0.454	-0.473	-0.338	-2.120	-2.291	-2.080	-1.960	-0.857	-0.865	-0.875	-0.770	-0.758	-0.677	-0.723	-0.568
243	-0.229	-0.408	-0.432	-0.288	-1.863	-2.046	-1.843	-1.696	-0.742	-0.781	-0.758	-0.678	-0.647	-0.581	-0.621	-0.514
243,5	-0.187	-0.385	-0.395	-0.250	-1.646	-1.829	-1.643	-1.467	-0.629	-0.724	-0.687	-0.601	-0.578	-0.493	-0.519	-0.478

244	-0.169	-0.382	-0.358	-0.232	-1.467	-1.636	-1.475	-1.277	-0.558	-0.670	-0.653	-0.559	-0.558	-0.424	-0.443	-0.466
244,5	-0.155	-0.373	-0.326	-0.212	-1.293	-1.467	-1.322	-1.123	-0.508	-0.607	-0.634	-0.516	-0.551	-0.356	-0.382	-0.456
245	-0.136	-0.351	-0.297	-0.171	-1.117	-1.321	-1.182	-0.979	-0.467	-0.551	-0.606	-0.476	-0.549	-0.305	-0.347	-0.434
245,5	-0.130	-0.317	-0.271	-0.147	-0.945	-1.189	-1.036	-0.855	-0.427	-0.510	-0.571	-0.431	-0.537	-0.265	-0.312	-0.392
246	-0.137	-0.277	-0.244	-0.119	-0.800	-1.065	-0.908	-0.734	-0.381	-0.479	-0.514	-0.369	-0.509	-0.232	-0.273	-0.337
246,5	-0.148	-0.245	-0.214	-0.094	-0.691	-0.945	-0.784	-0.609	-0.321	-0.449	-0.452	-0.311	-0.476	-0.212	-0.234	-0.263
247	-0.135	-0.220	-0.180	-0.059	-0.594	-0.825	-0.663	-0.464	-0.261	-0.406	-0.384	-0.254	-0.436	-0.179	-0.178	-0.168
247,5	-0.100	-0.191	-0.143	-0.009	-0.481	-0.707	-0.541	-0.314	-0.187	-0.344	-0.312	-0.198	-0.381	-0.121	-0.119	-0.065
248	-0.066	-0.175	-0.100	0.020	-0.375	-0.594	-0.424	-0.190	-0.142	-0.288	-0.253	-0.150	-0.340	-0.079	-0.088	-0.002
248,5	-0.034	-0.164	-0.063	0.025	-0.280	-0.496	-0.322	-0.104	-0.123	-0.248	-0.230	-0.121	-0.297	-0.060	-0.097	0.024
249	-0.005	-0.160	-0.039	0.030	-0.191	-0.421	-0.236	-0.052	-0.120	-0.220	-0.203	-0.091	-0.260	-0.071	-0.113	0.007
249,5	-0.013	-0.166	-0.033	0.014	-0.135	-0.367	-0.181	-0.040	-0.129	-0.227	-0.212	-0.074	-0.229	-0.106	-0.136	-0.024
250	-0.039	-0.178	-0.043	0.004	-0.110	-0.332	-0.166	-0.050	-0.158	-0.247	-0.215	-0.064	-0.223	-0.152	-0.146	-0.063
250,5	-0.060	-0.184	-0.066	0.004	-0.095	-0.314	-0.184	-0.056	-0.172	-0.257	-0.221	-0.062	-0.211	-0.173	-0.141	-0.077
251	-0.065	-0.190	-0.088	0.010	-0.081	-0.304	-0.198	-0.043	-0.160	-0.245	-0.190	-0.068	-0.193	-0.164	-0.126	-0.061
251,5	-0.053	-0.185	-0.099	0.021	-0.062	-0.295	-0.195	-0.019	-0.125	-0.224	-0.163	-0.064	-0.166	-0.126	-0.101	-0.013
252	-0.040	-0.186	-0.091	0.015	-0.059	-0.277	-0.183	-0.001	-0.086	-0.208	-0.134	-0.067	-0.148	-0.097	-0.106	0.016
252,5	-0.045	-0.184	-0.068	0.000	-0.064	-0.249	-0.156	0.012	-0.047	-0.200	-0.100	-0.068	-0.137	-0.075	-0.125	0.021
253	-0.022	-0.162	-0.035	-0.003	-0.050	-0.210	-0.107	0.051	0.009	-0.175	-0.049	-0.051	-0.123	-0.050	-0.138	0.033
253,5	-0.011	-0.144	-0.004	0.010	-0.022	-0.161	-0.066	0.078	0.054	-0.165	-0.015	-0.044	-0.130	-0.044	-0.145	0.025
254	0.008	-0.122	0.015	0.025	0.007	-0.113	-0.028	0.108	0.089	-0.149	0.000	-0.034	-0.127	-0.028	-0.135	0.023
254,5	0.023	-0.097	0.019	0.045	0.042	-0.077	-0.011	0.116	0.110	-0.135	0.001	-0.036	-0.156	-0.016	-0.111	0.021
255	0.046	-0.074	0.016	0.047	0.071	-0.056	0.000	0.116	0.120	-0.115	-0.015	-0.036	-0.188	-0.013	-0.091	0.030
255,5	0.054	-0.059	0.013	0.056	0.075	-0.049	0.003	0.094	0.125	-0.107	-0.030	-0.046	-0.218	-0.030	-0.070	0.044
256	0.078	-0.040	0.019	0.072	0.074	-0.049	0.020	0.099	0.121	-0.083	-0.028	-0.031	-0.216	-0.034	-0.044	0.072
256,5	0.105	-0.017	0.040	0.106	0.084	-0.047	0.052	0.125	0.115	-0.062	-0.003	0.004	-0.201	-0.028	-0.012	0.104
257	0.141	0.013	0.067	0.160	0.121	-0.038	0.110	0.179	0.109	-0.028	0.040	0.057	-0.158	0.003	0.025	0.144

257,5	0.173	0.032	0.091	0.202	0.147	-0.021	0.166	0.234	0.112	0.008	0.074	0.116	-0.115	0.032	0.054	0.176
258	0.180	0.026	0.100	0.203	0.164	-0.002	0.186	0.270	0.098	0.026	0.082	0.159	-0.093	0.059	0.045	0.183
258,5	0.178	-0.008	0.090	0.159	0.153	0.014	0.162	0.272	0.080	0.027	0.049	0.177	-0.095	0.063	0.006	0.163
259	0.157	-0.047	0.068	0.099	0.143	0.020	0.125	0.228	0.061	0.018	0.012	0.165	-0.102	0.046	-0.041	0.132
259,5	0.134	-0.092	0.043	0.028	0.116	0.022	0.075	0.175	0.050	-0.007	-0.022	0.143	-0.113	0.014	-0.092	0.097
260	0.119	-0.126	0.021	-0.021	0.112	0.019	0.032	0.130	0.049	-0.021	-0.042	0.115	-0.116	-0.001	-0.122	0.075

Dynamic Light Scattering (DLS)

Radius (nm)	IBMP-8.1				IBMP-8.2				IBMP-8.3				IBMP-8.4			
	Time of exposure (hours)				Time of exposure (hours)				Time of exposure (hours)				Time of exposure (hours)			
	**0**	**24**	**48**	**72**	**0**	**24**	**48**	**72**	**0**	**24**	**48**	**72**	**0**	**24**	**48**	**72**
0.0260057	0	0	0	0	0	0	0	0	0	0	0	0	0	0	0	0
0.0329794	0	0	0	0	0	0	0	0	0	0	0	0	0	0	0	0
0.0418231	0	0	0	0	0	0	0	0	0	0	0	0	0	0	0	0
0.0530384	0	0	0	0	0	0	0	0	0	0	0	0	0	0	0	0
0.0672611	0	0	0	0	0	0	0	0	0	0	0	0	0	0	0	0
0.0852978	0	0	0	0	0	0	0	0	0	0	0	0	0	0	0	0
0.108171	0	0	0	0	0	0	0	0	0	0	0	0	0	0	0	0
0.137178	0	0	0	0	0	0	0	0	0	0	0	0	0	0	0	0
0.173964	0	0	0	0	0	0	0	0	0	0	0	0	0	0	0	0
0.220614	0	0	0	0	0	0	0	0	0	0	0	0	0	0	0	0
0.279774	0	0	0	0	0	0	0	0	0	0	0	0	0	0	0	0
0.354797	0	0	0	0	0	0	0	0	0	0	0	0	0	0	0	0
0.44994	0	0	0	0	0	0	0	0	0	0	0	0	0	0	0	0
0.570595	0	0	0	0	0	0	0	0	0	0	0	0	0	0	0	0
0.723605	0	0	0	0	0	0	0	0	0	0	0	0	0	0	0	0
0.917647	0	0	0	0	0	0	0	0	0	0	0	0	0	0	0	0
1.16372	0	0	0	0	0	0	0	0	0	0	0	0	0	0	0	0
1.47579	0	0	0	6.937	0	0	0	0	0	0	0	0	0	0	0	0
1.87153	5.405	0	10.983	12.595	0	0	0	0	0	0	0	0	0	0	0	0
2.3734	48.895	32.038	33.009	27.779	0	0	17.722	9.759	0	13.662	0	0	0	0	0	4.953
3.00985	30.414	36.008	29.595	25.955	47.823	39.251	38.342	40.559	9.713	34.260	33.601	0	21.356	32.796	30.976	25.539

3.81697	9.252	18.180	14.628	13.375	36.099	41.643	26.881	30.047	73.570	26.637	36.679	33.728	34.049	31.924	31.056	25.969
4.84052	1.493	4.594	4.424	4.192	13.631	16.034	11.813	12.666	22.038	14.255	19.776	19.800	22.686	18.962	18.846	16.750
6.13855	0	0	0.713	0.693	2.328	1.530	3.435	3.013	4.794	6.257	6.860	3.882	10.139	8.606	8.662	8.156
7.78466	0	0	0	0.221	0	0	0.496	0.080	0	2.371	1.398	0	3.276	3.154	3.165	3.073
9.87219	0	0	0	0	0	0	0	0	0	0	0	0	0.678	0.917	0.886	0.829
12.5195	0	0	0	0	0	0	0	0	0	0	0	0	0	0	0	0
15.8767	0	0	0	0	0	0	0	0	0	0	0	0	0	0	0	0
20.1342	0	0	0	0	0	0	0	0	0	0	0	0	0	0	0	0
25.5334	0	0	0	0	0	0	0	0	0	0	0	0	0	0	0	0
32.3804	0	0	0	0	0	0	0	0	0	0	0	0	0	0	0	0
41.0635	0	0	0	0	0	0	0	0	0	0	0	0	0	0	0	0
52.0751	0	0	0	0	0	0	0	0	0	0	0	0	0	0	0	0
66.0395	0	0	0	0	0	0	0	0	0	0	0	0	0	0	0	0
83.7486	0	0	0	0	0	0	0	0	0	0	0	0	0	0	0	0
106.207	0	0	0	0	0	0	0	0	0	0	0	0	0	0	0	0
134.687	0	0	0	0	0	0	0	0	0	0	0	0	0	0	0	0
170.804	0	0	0	0	0	0	0	0	0	0	0	0	0	0	0	0
216.607	0	0	0	0	0	0	0	0	0	0	0	0	0	0	0	0
274.692	0	0	0	0	0	0	0	0	0	0	0	0	0	0	0	0
348.354	0	0	0	0	0	0	0	0	0	0	0	0	0	0	0	0
441.768	0	0	0	0	0	0	0	0	0	0	0	0	0	0	0	0
560.232	0	0	0	0	0	0	0	0	0	0	0	0	0	0	0	0
710.463	0	0	0	0	0	0	0	0	0	0	0	0	0	0	0	0
900.981	0	0	0	0	0	0	0	0	0	0	0	0	0	0	0	0
1142.59	0	0	0	0	0	0	0	0	0	0	0	0	0	0	0	0
1448.98	0	0	0	0	0	0	0	0	0	0	0	0	0	0	0	0
1837.54	0	0	0	0	0	0	0	0	0	0	0	0.038	0	0	0	0

2330.29	0	0	0	0	0	0	0	0	0	0	0	0.093	0	0	0	0
2955.18	0	0	0	0	0	0	0	0	0	0	0	0.174	0	0	0	0
3747.64	0	0	0	0	0	0	0	0	0	0	0	39.586	0	0	0	0
4752.61	0	0	0	0	0	0	0	0	0	0	0	0.262	0	0	0	0
6027.06	0	0	0	0	0	0	0	0	0	0	0	0.288	0	0	0	0
7643.28	0	0	0	0	0	0	0	0	0	0	0	0.578	0	0	0	0
9692.89	0	0	0	0	0	0	0	0	0	0	0	0.304	0	0	0	0
12292.1	0	0	0	0	0	0	0	0	0	0	0	0	0	0	0	0

Indirect ELISA

Samples	IBMP-8.1				IBMP-8.2				IBMP-8.3				IBMP-8.4			
	Time of exposure (hours)				Time of exposure (hours)				Time of exposure (hours)				Time of exposure (hours)			
	**0**	**24**	**48**	**72**	**0**	**24**	**48**	**72**	**0**	**24**	**48**	**72**	**0**	**24**	**48**	**72**
Trypanosoma cruzi-negative 01	1.11	0.66	0.69	0.37	0.27	0.25	0.27	0.31	0.36	0.60	0.44	0.29	0.53	0.28	0.44	0.35
Trypanosoma cruzi-negative 02	2.43	1.69	1.97	0.92	0.28	0.17	0.20	0.38	0.19	0.32	0.23	0.25	0.34	0.23	0.25	0.24
Trypanosoma cruzi-negative 03	1.90	0.72	0.71	0.38	0.29	0.18	0.32	0.29	0.23	0.32	0.53	0.29	0.29	0.28	0.33	0.32
Trypanosoma cruzi-negative 04	2.09	1.08	1.25	0.48	0.21	0.16	0.18	0.23	0.17	0.21	0.18	0.33	0.38	0.25	0.24	0.29
Trypanosoma cruzi-negative 05	0.77	0.57	0.72	0.61	0.25	0.16	0.21	0.28	0.73	0.37	0.69	0.49	0.27	0.18	0.22	0.28
Trypanosoma cruzi-negative 06	1.28	0.67	0.99	0.48	0.28	0.20	0.22	0.24	0.20	0.38	0.29	0.29	0.21	0.18	0.26	0.23
Trypanosoma cruzi-negative 07	1.11	0.79	0.79	0.52	0.24	0.19	0.20	0.23	0.21	0.24	0.23	0.24	0.27	0.19	0.21	0.22
Trypanosoma cruzi-negative 08	1.86	0.84	0.87	0.46	0.23	0.23	0.20	0.34	0.25	0.40	0.27	0.26	0.21	0.18	0.23	0.24
Trypanosoma cruzi-negative 09	0.74	0.65	0.74	0.37	0.27	0.20	0.26	0.27	0.21	0.64	0.30	0.29	0.29	0.28	0.26	0.28
Trypanosoma cruzi-negative 10	0.91	1.30	1.45	0.68	0.22	0.25	0.25	0.30	0.25	0.30	0.28	0.54	0.34	0.27	0.27	0.36
Trypanosoma cruzi-negative 11	1.89	1.13	1.28	0.68	0.49	0.25	0.24	0.34	0.21	0.32	0.31	0.31	0.38	0.19	0.27	0.27
Trypanosoma cruzi-negative 12	1.42	0.88	0.86	0.35	0.23	0.18	0.25	0.34	0.20	0.35	0.35	0.30	0.56	0.40	0.28	0.40
Trypanosoma cruzi-negative 13	0.64	0.58	0.54	0.32	0.27	0.24	0.58	0.40	0.30	0.44	0.45	0.43	0.44	0.28	0.40	0.25
Trypanosoma cruzi-negative 14	1.33	0.62	0.71	0.50	0.22	0.17	0.34	0.24	0.18	0.24	0.24	0.25	0.19	0.17	0.23	0.22
Trypanosoma cruzi-negative 15	0.61	0.33	0.36	0.25	0.21	0.21	0.19	0.20	0.20	0.29	0.25	0.26	0.21	0.20	1.57	0.24
Trypanosoma cruzi-negative 16	2.20	1.30	1.26	0.64	0.23	0.24	0.27	0.29	0.20	0.25	0.27	0.28	0.19	0.20	0.25	0.26
Trypanosoma cruzi-negative 17	1.52	0.45	0.50	0.31	0.24	0.18	0.22	0.23	0.24	0.38	0.28	0.25	0.36	0.19	0.28	0.24
Trypanosoma cruzi-negative 18	1.18	0.86	0.89	0.55	0.17	0.22	0.18	0.27	0.22	0.23	0.24	0.21	0.21	0.16	0.15	0.20
Trypanosoma cruzi-negative 19	1.64	0.73	0.92	0.41	0.30	0.16	0.20	0.26	0.18	0.31	0.46	0.25	0.24	0.15	0.18	0.20
Trypanosoma cruzi-negative 20	1.16	0.72	0.94	0.38	0.29	0.20	0.25	0.27	0.23	0.31	0.24	0.36	0.35	0.25	0.24	0.30
Trypanosoma cruzi-negative 21	0.68	0.34	0.42	0.43	0.37	0.25	0.28	0.31	0.24	0.35	0.32	0.27	0.36	0.26	0.29	0.26

Trypanosoma cruzi-negative 22	2.11	1.18	1.56	0.66	0.22	0.45	0.19	0.23	0.21	0.33	0.32	0.25	0.19	0.17	0.25	0.25
Trypanosoma cruzi-negative 23	2.65	1.51	1.95	0.80	0.35	0.22	0.21	0.29	0.23	0.31	0.26	0.29	0.22	0.21	0.26	0.28
Trypanosoma cruzi-negative 24	0.55	0.33	0.51	0.26	0.30	0.29	0.29	0.43	0.25	0.39	0.35	0.50	0.33	0.31	0.31	0.32
Trypanosoma cruzi-negative 25	0.55	0.43	0.58	0.28	0.22	0.22	0.43	0.31	0.21	0.25	0.28	0.34	0.23	0.18	0.24	0.24
Trypanosoma cruzi-negative 26	1.41	0.50	0.75	0.29	0.30	0.18	0.19	0.32	0.24	0.25	0.23	0.28	0.20	0.18	0.21	0.21
Trypanosoma cruzi-negative 27	1.74	1.00	0.95	0.48	0.27	0.22	0.26	0.34	0.25	0.30	0.46	0.25	0.24	0.26	0.27	0.28
Trypanosoma cruzi-negative 28	1.44	0.59	0.68	0.29	0.26	0.23	0.31	0.57	0.30	0.45	0.67	0.32	0.61	0.40	0.42	0.34
Trypanosoma cruzi-negative 29	1.65	0.74	0.77	0.42	0.18	0.16	0.21	0.21	0.18	0.28	0.38	0.25	0.23	0.17	0.27	0.20
Trypanosoma cruzi-negative 30	1.76	0.64	1.00	0.44	0.24	0.22	0.23	0.30	0.22	0.29	0.28	0.25	0.22	0.22	0.28	0.28
Trypanosoma cruzi-negative 31	1.52	0.52	0.56	0.22	0.32	0.23	0.20	0.33	0.22	0.30	0.27	0.23	0.21	0.22	0.28	0.24
Trypanosoma cruzi-negative 32	1.90	1.00	0.88	0.46	0.22	0.17	0.23	0.24	0.17	0.38	0.26	0.26	0.16	0.16	0.27	0.28
Trypanosoma cruzi-negative 33	0.87	0.56	0.73	0.33	0.20	0.17	0.19	0.25	0.16	0.19	0.21	0.23	0.21	0.15	0.23	0.25
Trypanosoma cruzi-negative 34	1.80	0.95	0.90	0.35	0.27	0.25	0.35	0.28	0.27	0.33	0.40	0.18	0.39	0.25	0.32	0.32
Trypanosoma cruzi-negative 35	1.56	1.27	1.92	0.55	0.27	0.27	0.31	0.34	0.28	0.36	0.38	0.12	0.47	0.23	0.38	0.32
Trypanosoma cruzi-negative 36	1.65	0.83	0.97	0.31	0.19	0.20	0.17	0.25	0.20	0.23	0.28	0.26	0.29	0.17	0.23	0.30
Trypanosoma cruzi-negative 37	0.92	0.38	0.47	0.25	0.19	0.21	0.22	0.43	0.20	0.28	0.26	0.40	0.36	0.25	0.25	0.27
Trypanosoma cruzi-negative 38	1.91	1.04	1.43	0.67	0.26	0.20	0.23	0.27	0.25	0.45	0.31	0.36	0.30	0.41	0.27	0.26
Trypanosoma cruzi-negative 39	2.32	1.41	1.67	0.84	0.29	0.33	0.31	0.40	0.27	0.50	0.38	0.18	0.36	0.28	0.34	0.37
Trypanosoma cruzi-negative 40	2.60	2.17	2.55	1.10	0.29	0.25	0.22	0.29	0.19	0.37	0.27	0.27	0.30	0.21	0.25	0.23
Trypanosoma cruzi-negative 41	1.18	0.67	0.82	0.32	0.22	0.19	0.24	0.25	0.20	0.26	0.30	0.27	0.31	0.20	0.35	0.30
Trypanosoma cruzi-negative 42	1.31	0.86	1.14	0.62	0.17	0.14	0.16	0.18	0.16	0.19	0.20	0.22	0.19	0.14	0.20	0.25
Trypanosoma cruzi-negative 43	0.95	0.74	0.90	0.44	0.23	0.16	0.24	0.29	0.19	0.28	0.28	0.30	0.21	0.16	0.28	0.27
Trypanosoma cruzi-negative 44	0.76	0.63	0.74	0.45	0.19	0.21	0.21	0.32	0.20	0.29	0.32	0.31	0.62	0.37	0.27	0.36
Trypanosoma cruzi-negative 45	1.46	1.04	1.22	0.65	0.17	0.19	0.22	0.29	0.19	0.40	0.22	0.27	0.29	0.20	0.25	0.25
Trypanosoma cruzi-negative 46	1.43	0.87	1.27	0.75	0.29	1.01	0.35	0.41	0.27	0.33	0.24	0.33	0.63	0.42	0.46	0.39
Trypanosoma cruzi-positive 01	0.28	0.27	0.32	0.31	1.04	0.66	0.82	0.79	1.81	2.44	1.95	1.16	1.26	0.62	0.60	0.54

Trypanosoma cruzi-positive 02	0.21	0.21	0.27	0.37	2.37	2.27	2.17	2.44	2.80	3.32	2.82	2.55	2.76	1.56	1.00	0.61
Trypanosoma cruzi-positive 03	0.24	0.28	0.31	0.33	2.00	1.99	1.55	1.75	2.49	2.99	2.95	2.25	2.19	0.88	0.60	0.53
Trypanosoma cruzi-positive 04	0.17	0.19	0.18	0.21	2.21	2.10	1.81	1.84	2.07	2.16	1.95	1.49	1.90	0.81	0.63	0.54
Trypanosoma cruzi-positive 05	0.14	0.24	0.22	0.34	0.82	0.62	0.69	0.85	1.35	2.03	1.41	1.16	1.15	0.58	0.50	0.49
Trypanosoma cruzi-positive 06	0.20	0.22	0.32	0.21	1.66	1.88	1.55	1.46	1.78	1.99	1.62	1.44	1.65	0.60	0.50	0.44
Trypanosoma cruzi-positive 07	0.21	0.28	0.26	0.26	1.19	1.38	1.09	1.04	1.35	1.02	1.40	1.04	1.48	0.96	0.61	0.58
Trypanosoma cruzi-positive 08	0.21	0.24	0.26	0.25	1.65	2.17	2.19	1.98	2.65	2.97	2.63	2.38	2.06	0.95	0.87	0.70
Trypanosoma cruzi-positive 09	0.21	0.23	0.30	0.27	0.88	1.10	0.82	1.33	1.65	1.77	1.97	1.41	1.26	0.40	0.31	0.24
Trypanosoma cruzi-positive 10	0.20	0.24	0.31	0.29	1.14	1.36	1.28	1.69	1.35	1.93	1.46	1.03	1.74	0.71	0.57	0.49
Trypanosoma cruzi-positive 11	0.24	0.21	0.27	0.37	1.55	1.84	1.34	1.84	1.63	1.60	1.51	1.27	1.90	1.32	1.03	0.83
Trypanosoma cruzi-positive 12	0.21	0.22	0.26	0.30	1.60	1.73	1.56	1.69	1.79	2.20	1.85	1.38	1.60	0.57	0.46	0.42
Trypanosoma cruzi-positive 13	0.39	0.33	0.60	0.45	0.66	0.53	0.65	0.62	0.93	1.60	0.97	0.65	0.88	0.56	0.45	0.34
Trypanosoma cruzi-positive 14	0.24	0.22	0.26	0.23	1.56	1.19	1.10	0.90	1.34	2.27	0.99	0.90	1.78	0.85	0.50	0.40
Trypanosoma cruzi-positive 15	0.23	0.24	0.34	0.22	0.74	0.48	0.45	0.31	0.91	0.92	0.70	0.53	0.73	0.29	0.33	0.28
Trypanosoma cruzi-positive 16	0.19	0.22	0.24	0.23	2.64	2.48	2.37	2.04	2.67	3.12	2.52	2.40	2.46	1.27	0.78	0.75
Trypanosoma cruzi-positive 17	0.21	0.24	0.27	0.30	0.91	0.40	0.57	0.68	1.99	2.84	2.13	1.32	1.83	0.62	0.55	0.40
Trypanosoma cruzi-positive 18	0.18	0.19	0.27	0.24	1.60	1.56	1.38	1.56	2.08	2.27	2.10	1.59	1.61	0.91	0.78	0.72
Trypanosoma cruzi-positive 19	0.20	0.16	0.22	0.19	1.14	1.05	0.90	0.98	0.93	1.37	0.86	0.69	1.70	0.61	0.50	0.41
Trypanosoma cruzi-positive 20	0.20	0.27	0.35	0.33	1.24	1.22	1.04	0.99	2.21	2.72	1.85	1.53	1.27	0.50	0.46	0.41
Trypanosoma cruzi-positive 21	0.24	0.27	0.33	0.21	0.80	0.59	0.48	0.46	0.48	0.85	0.61	0.42	0.95	0.35	0.27	0.27
Trypanosoma cruzi-positive 22	0.24	0.23	0.27	0.25	2.32	2.59	2.38	2.17	2.66	3.11	2.59	2.47	2.73	1.44	0.93	0.63
Trypanosoma cruzi-positive 23	0.25	0.25	0.30	0.24	2.01	2.37	2.45	2.15	2.65	3.01	2.63	2.11	2.46	1.68	1.24	0.80
Trypanosoma cruzi-positive 24	0.27	0.31	0.42	0.34	1.44	1.19	1.15	0.73	1.74	2.27	1.33	1.11	1.43	0.36	0.32	0.29
Trypanosoma cruzi-positive 25	0.19	0.26	0.31	0.23	0.68	0.72	0.75	1.03	0.58	1.03	0.68	0.40	0.76	0.33	0.30	0.24
Trypanosoma cruzi-positive 26	0.19	0.19	0.50	0.28	1.49	1.63	1.23	1.63	2.53	2.97	2.43	1.87	2.00	1.17	0.76	0.51
Trypanosoma cruzi-positive 27	0.24	0.26	0.32	0.34	1.90	1.92	1.64	1.86	2.42	2.65	2.54	2.06	1.96	1.06	0.85	0.53
Trypanosoma cruzi-positive 28	0.23	0.33	0.39	0.46	1.46	0.99	1.08	0.94	1.66	2.46	1.82	1.38	1.61	0.45	0.36	0.26

Trypanosoma cruzi-positive 29	0.27	0.26	0.40	0.18	2.39	2.05	1.72	1.58	2.16	2.98	2.43	2.05	2.45	0.88	0.58	0.39
Trypanosoma cruzi-positive 30	0.25	0.24	0.40	0.26	2.38	2.66	1.80	2.19	2.68	3.11	2.48	2.37	2.52	1.39	0.83	0.52
Trypanosoma cruzi-positive 31	0.25	0.24	0.32	0.22	1.77	1.67	1.37	1.25	2.37	3.04	2.20	1.75	2.05	0.57	0.31	0.24
Trypanosoma cruzi-positive 32	0.19	0.18	0.31	0.20	2.32	2.36	2.08	1.87	2.55	3.03	2.22	1.94	2.36	0.94	0.66	0.35
Trypanosoma cruzi-positive 33	0.19	0.19	0.30	0.20	1.12	1.01	1.01	1.00	1.41	2.03	1.67	1.16	1.39	0.48	0.43	0.32
Trypanosoma cruzi-positive 34	0.26	0.27	0.47	0.30	1.52	1.27	1.06	0.83	1.51	2.16	1.36	1.02	1.49	0.75	0.54	0.44
Trypanosoma cruzi-positive 35	0.34	0.32	0.42	0.32	1.25	1.18	0.93	1.02	2.19	2.79	2.02	1.95	1.77	0.74	0.52	0.45
Trypanosoma cruzi-positive 36	0.22	0.18	0.23	0.19	1.68	2.18	1.68	1.85	2.16	2.78	2.14	2.00	2.08	0.66	0.37	0.35
Trypanosoma cruzi-positive 37	0.20	0.23	0.34	0.17	0.97	1.15	0.76	0.84	2.30	2.94	2.33	1.92	1.64	0.70	0.39	0.33
Trypanosoma cruzi-positive 38	0.22	0.23	0.27	0.21	1.38	1.77	1.07	1.38	2.31	2.74	2.26	1.98	2.10	1.35	0.63	0.56
Trypanosoma cruzi-positive 39	0.26	0.65	0.19	0.32	1.94	1.86	1.64	1.54	2.73	2.96	2.44	2.21	2.34	0.75	0.60	0.45
Trypanosoma cruzi-positive 40	0.18	0.20	0.34	0.19	1.72	2.46	2.24	2.18	2.54	3.15	2.47	2.33	3.01	1.54	1.09	0.33
Trypanosoma cruzi-positive 41	0.24	0.21	0.26	0.20	1.53	0.92	1.04	0.84	1.74	2.59	1.59	1.22	1.44	0.36	0.38	0.16
Trypanosoma cruzi-positive 42	0.17	0.25	0.28	0.18	1.80	1.57	1.37	1.45	2.59	3.11	1.66	1.84	1.83	1.08	0.99	0.79
Trypanosoma cruzi-positive 43	0.21	0.22	0.34	0.21	1.90	2.19	1.90	1.93	2.09	3.22	2.39	1.88	1.71	0.92	0.74	0.64
Trypanosoma cruzi-positive 44	0.21	0.15	0.29	0.21	0.93	1.05	0.81	0.96	1.32	2.16	1.15	0.91	1.32	1.04	0.83	0.55
Trypanosoma cruzi-positive 45	0.21	0.19	0.32	0.20	1.43	1.33	1.37	1.37	2.15	3.11	2.54	1.77	1.89	0.76	0.73	0.54
Trypanosoma cruzi-positive 46	0.28	0.25	0.47	0.32	1.92	1.74	1.96	1.81	2.40	3.30	2.43	2.33	1.84	1.51	0.89	0.50

Indirect ELISA – IBMP-8.1

Samples	IBMP-8.1							
	Time of exposure (DAYS)							
	**0**	**52**	**104**	**156**	**206**	**260**	**312**	**364**
Trypanosoma cruzi-negative 01	0.24	0.27	0.29	0.19	0.19	0.23	0.38	0.33
Trypanosoma cruzi-negative 02	0.36	0.34	0.36	0.23	0.23	0.31	0.65	0.23
Trypanosoma cruzi-negative 03	0.26	0.17	0.25	0.25	0.23	0.24	0.69	0.22
Trypanosoma cruzi-negative 04	0.30	0.29	0.27	0.31	0.22	0.21	0.40	0.27
Trypanosoma cruzi-negative 05	0.27	0.27	0.26	0.27	0.27	0.25	0.31	0.51
Trypanosoma cruzi-negative 06	0.43	0.37	0.11	0.33	0.28	0.33	0.49	0.30
Trypanosoma cruzi-negative 07	0.29	0.08	0.32	0.20	0.26	0.28	0.34	0.38
Trypanosoma cruzi-negative 08	0.32	0.16	0.32	0.22	0.26	0.34	0.49	0.25
Trypanosoma cruzi-negative 09	0.19	0.23	0.20	0.29	0.26	0.22	0.34	0.17
Trypanosoma cruzi-negative 10	0.30	0.36	0.31	0.37	0.24	0.30	0.54	0.26
Trypanosoma cruzi-negative 11	0.28	0.29	0.28	0.25	0.21	0.24	0.24	0.24
Trypanosoma cruzi-negative 12	0.38	0.38	0.37	0.30	0.31	0.47	0.48	0.33
Trypanosoma cruzi-negative 13	0.33	0.31	0.30	0.30	0.24	0.22	0.52	0.29
Trypanosoma cruzi-negative 14	0.22	0.25	0.23	0.29	0.22	0.24	0.28	0.19
Trypanosoma cruzi-negative 15	0.37	0.35	0.33	0.30	0.30	0.35	0.50	0.48
Trypanosoma cruzi-negative 16	0.31	0.30	0.35	0.29	0.26	0.29	0.28	0.24
Trypanosoma cruzi-negative 17	0.27	0.30	0.35	0.54	0.34	0.31	0.48	0.44
Trypanosoma cruzi-negative 18	0.29	0.07	0.32	0.23	0.27	0.25	0.61	0.25
Trypanosoma cruzi-negative 19	0.24	0.27	0.27	0.34	0.26	0.26	0.43	0.26
Trypanosoma cruzi-negative 20	0.31	0.30	0.29	0.23	0.25	0.21	0.41	0.30

Trypanosoma cruzi-negative 21	0.39	0.08	0.39	0.15	0.30	0.40	0.29	0.30
Trypanosoma cruzi-negative 22	0.44	0.35	0.35	0.27	0.25	0.29	0.49	0.27
Trypanosoma cruzi-negative 23	0.37	0.37	0.38	0.27	0.23	0.24	0.49	0.32
Trypanosoma cruzi-negative 24	0.27	0.27	0.26	0.30	0.26	0.21	0.39	0.23
Trypanosoma cruzi-negative 25	0.28	0.30	0.24	0.45	0.29	0.19	0.48	0.26
Trypanosoma cruzi-negative 26	0.18	0.24	0.22	0.42	0.27	0.20	0.49	0.42
Trypanosoma cruzi-negative 27	0.29	0.07	0.30	0.25	0.26	0.30	0.44	0.28
Trypanosoma cruzi-negative 28	0.27	0.07	0.30	0.23	0.27	0.30	0.43	0.27
Trypanosoma cruzi-negative 29	0.34	0.17	0.17	0.21	0.26	0.31	0.45	0.29
Trypanosoma cruzi-negative 30	0.34	0.40	0.35	0.22	0.35	0.26	0.42	0.29
Trypanosoma cruzi-negative 31	0.15	0.08	0.16	0.47	0.20	0.22	0.37	0.14
Trypanosoma cruzi-negative 32	0.36	0.36	0.35	0.30	0.39	0.21	0.44	0.33
Trypanosoma cruzi-negative 33	0.21	0.17	0.22	0.24	0.45	0.47	0.32	0.20
Trypanosoma cruzi-negative 34	0.32	0.07	0.32	0.22	0.30	0.31	0.44	0.33
Trypanosoma cruzi-negative 35	0.38	0.35	0.36	0.23	0.25	0.27	0.50	0.31
Trypanosoma cruzi-negative 36	0.33	0.34	0.39	0.29	0.26	0.23	1.03	0.27
Trypanosoma cruzi-negative 37	0.26	0.26	0.26	0.17	0.29	0.21	0.60	0.23
Trypanosoma cruzi-negative 38	0.38	0.33	0.33	0.29	0.23	0.27	0.42	0.29
Trypanosoma cruzi-negative 39	0.30	0.29	0.27	0.21	0.24	0.29	0.42	0.22
Trypanosoma cruzi-negative 40	0.31	0.26	0.29	0.31	0.25	0.24	0.35	0.32
Trypanosoma cruzi-negative 41	0.38	0.34	0.37	0.29	0.31	0.32	0.46	0.55
Trypanosoma cruzi-negative 42	0.31	0.30	0.27	0.26	0.30	0.24	0.42	0.49
Trypanosoma cruzi-negative 43	0.37	0.32	0.34	0.27	0.26	0.23	0.69	0.27
Trypanosoma cruzi-negative 44	0.29	0.33	0.31	0.51	0.31	0.22	0.48	0.27
Trypanosoma cruzi-negative 45	0.28	0.27	0.25	0.31	0.39	0.26	0.45	0.26

Trypanosoma cruzi-negative 46	0.20	0.24	0.21	0.33	0.46	0.21	0.44	0.48
Trypanosoma cruzi-positive 01	1.74	1.50	1.45	0.45	1.29	1.19	1.35	1.37
Trypanosoma cruzi-positive 02	0.62	0.41	0.35	0.92	0.51	0.35	0.49	0.30
Trypanosoma cruzi-positive 03	0.60	0.40	0.37	0.18	1.56	0.34	0.35	0.30
Trypanosoma cruzi-positive 04	2.13	1.73	1.56	0.55	1.15	1.40	1.78	1.47
Trypanosoma cruzi-positive 05	0.67	1.13	1.09	2.17	1.41	0.89	1.18	0.91
Trypanosoma cruzi-positive 06	2.49	2.23	1.92	2.42	0.88	1.62	2.21	1.80
Trypanosoma cruzi-positive 07	0.74	0.68	0.63	2.01	0.46	0.48	0.93	0.59
Trypanosoma cruzi-positive 08	2.53	1.97	1.87	2.30	1.24	1.58	2.02	1.66
Trypanosoma cruzi-positive 09	2.09	2.18	1.77	1.77	0.81	1.30	2.02	1.84
Trypanosoma cruzi-positive 10	0.87	0.79	0.70	1.18	1.24	0.66	0.88	0.64
Trypanosoma cruzi-positive 11	2.31	2.01	1.79	1.52	1.24	1.58	1.93	1.81
Trypanosoma cruzi-positive 12	1.15	1.19	1.09	0.38	1.41	0.88	1.26	1.02
Trypanosoma cruzi-positive 13	1.68	1.56	1.47	0.93	1.26	1.42	1.72	1.34
Trypanosoma cruzi-positive 14	2.03	1.83	1.66	2.87	1.49	1.65	1.99	1.60
Trypanosoma cruzi-positive 15	2.63	2.46	2.12	0.78	1.65	2.11	2.25	2.10
Trypanosoma cruzi-positive 16	2.07	2.05	1.74	1.19	0.45	0.46	1.91	1.73
Trypanosoma cruzi-positive 17	1.37	1.29	1.09	0.92	1.14	0.92	1.07	1.00
Trypanosoma cruzi-positive 18	1.40	1.26	1.21	1.61	0.92	0.89	1.28	1.09
Trypanosoma cruzi-positive 19	1.39	1.20	1.04	1.49	1.03	0.83	1.23	1.79
Trypanosoma cruzi-positive 20	1.16	1.02	1.01	1.63	1.19	0.88	1.14	0.98
Trypanosoma cruzi-positive 21	2.09	0.37	1.82	1.57	1.82	1.59	1.34	0.92
Trypanosoma cruzi-positive 22	2.15	2.01	1.59	2.75	0.98	1.39	1.60	1.65
Trypanosoma cruzi-positive 23	3.03	2.55	2.26	1.04	1.91	2.25	2.55	1.64
Trypanosoma cruzi-positive 24	2.74	2.36	2.14	2.03	2.12	1.99	2.58	2.30

Trypanosoma cruzi-positive 25	2.27	2.09	1.85	1.74	1.64	1.65	2.09	1.23
Trypanosoma cruzi-positive 26	2.28	2.16	1.81	0.98	1.68	1.89	2.13	1.99
Trypanosoma cruzi-positive 27	2.19	2.19	1.97	1.57	2.03	1.85	2.00	1.87
Trypanosoma cruzi-positive 28	1.20	1.16	1.07	1.12	1.85	0.83	1.03	0.98
Trypanosoma cruzi-positive 29	0.72	0.82	0.75	0.97	0.90	0.53	0.91	0.57
Trypanosoma cruzi-positive 30	1.19	1.49	0.97	0.50	1.12	0.80	1.25	0.91
Trypanosoma cruzi-positive 31	2.72	2.53	2.03	2.07	1.95	2.11	2.33	2.13
Trypanosoma cruzi-positive 32	0.71	0.64	1.55	1.72	1.59	0.41	0.65	0.37
Trypanosoma cruzi-positive 33	1.31	1.20	1.25	1.69	1.14	0.95	1.17	0.86
Trypanosoma cruzi-positive 34	3.07	0.92	2.36	2.45	1.80	2.20	2.74	2.20
Trypanosoma cruzi-positive 35	2.48	2.23	1.90	1.72	1.24	1.65	1.94	1.91
Trypanosoma cruzi-positive 36	0.56	0.31	0.35	1.75	1.85	0.40	0.58	0.28
Trypanosoma cruzi-positive 37	2.69	2.69	2.11	2.58	2.12	2.17	2.35	1.23
Trypanosoma cruzi-positive 38	1.19	1.25	1.02	0.63	1.26	0.86	0.89	0.95
Trypanosoma cruzi-positive 39	0.66	0.54	0.46	1.49	1.63	0.41	0.61	0.40
Trypanosoma cruzi-positive 40	1.46	1.06	0.75	1.83	0.96	0.75	1.04	0.89
Trypanosoma cruzi-positive 41	2.14	2.05	1.69	0.99	1.55	1.43	2.10	1.55
Trypanosoma cruzi-positive 42	3.15	0.92	2.41	1.78	2.18	2.38	3.05	1.46
Trypanosoma cruzi-positive 43	1.33	1.19	0.97	2.39	1.19	0.52	1.14	0.89
Trypanosoma cruzi-positive 44	2.08	1.41	1.44	1.41	1.69	1.24	1.75	1.43
Trypanosoma cruzi-positive 45	1.41	1.40	1.20	1.99	1.70	0.84	1.57	1.21
Trypanosoma cruzi-positive 46	2.09	1.96	1.59	1.97	2.28	1.23	1.64	1.62

Indirect ELISA – IBMP-8.2

Samples	IBMP-8.2 Time of exposure (DAYS)							
	0	52	104	156	206	260	312	364
*Trypanosoma cruz*i-negative 01	0.36	0.36	0.27	0.39	0.39	0.20	0.39	0.37
Trypanosoma cruzi-negative 02	0.48	0.45	0.34	0.40	0.36	0.31	0.62	0.57
Trypanosoma cruzi-negative 03	0.40	0.33	0.28	0.27	0.26	0.25	0.92	0.17
Trypanosoma cruzi-negative 04	0.37	0.31	0.29	0.30	0.28	0.27	0.38	0.16
Trypanosoma cruzi-negative 05	0.36	0.32	0.23	0.25	0.23	0.22	0.48	0.15
Trypanosoma cruzi-negative 06	0.63	0.48	0.40	0.33	0.31	0.29	0.72	0.25
Trypanosoma cruzi-negative 07	0.45	0.39	0.29	0.29	0.28	0.28	0.53	0.18
Trypanosoma cruzi-negative 08	0.38	0.31	0.27	0.31	0.30	0.29	0.37	0.15
Trypanosoma cruzi-negative 09	0.25	0.22	0.17	0.23	0.22	0.21	0.34	0.12
Trypanosoma cruzi-negative 10	0.39	0.32	0.26	0.32	0.30	0.27	0.49	0.24
Trypanosoma cruzi-negative 11	0.50	0.39	0.24	0.32	0.27	0.21	0.56	0.18
Trypanosoma cruzi-negative 12	0.50	0.42	0.32	0.37	0.34	0.32	0.48	0.19
Trypanosoma cruzi-negative 13	0.42	0.39	0.29	0.27	0.27	0.27	0.45	0.50
Trypanosoma cruzi-negative 14	0.35	0.27	0.20	0.24	0.24	0.25	0.20	0.53
Trypanosoma cruzi-negative 15	0.63	0.51	0.46	0.44	0.43	0.42	0.72	0.23
Trypanosoma cruzi-negative 16	0.38	0.36	0.30	0.28	0.27	0.26	0.48	0.17
Trypanosoma cruzi-negative 17	0.49	0.37	0.28	0.29	0.28	0.27	0.51	0.24
Trypanosoma cruzi-negative 18	0.32	0.32	0.26	0.27	0.25	0.24	0.36	0.19
Trypanosoma cruzi-negative 19	0.38	0.30	0.25	0.24	0.23	0.23	0.37	0.42
Trypanosoma cruzi-negative 20	0.44	0.30	0.27	0.28	0.26	0.25	0.64	0.56

Trypanosoma cruzi-negative 21	0.48	0.40	0.34	0.37	0.34	0.31	0.60	0.58
Trypanosoma cruzi-negative 22	0.39	0.33	0.27	0.31	0.27	0.23	0.42	0.18
Trypanosoma cruzi-negative 23	0.55	0.47	0.37	0.39	0.37	0.34	0.55	0.45
Trypanosoma cruzi-negative 24	0.33	0.28	0.23	0.24	0.23	0.22	0.38	0.14
Trypanosoma cruzi-negative 25	0.43	0.29	0.21	0.26	0.23	0.20	0.39	0.16
Trypanosoma cruzi-negative 26	0.23	0.24	0.15	0.22	0.20	0.17	0.31	0.42
Trypanosoma cruzi-negative 27	0.44	0.39	0.32	0.32	0.30	0.28	0.48	0.58
Trypanosoma cruzi-negative 28	0.30	0.31	0.24	0.28	0.26	0.24	0.24	0.34
Trypanosoma cruzi-negative 29	0.44	0.39	0.29	0.38	0.32	0.27	0.54	0.21
Trypanosoma cruzi-negative 30	0.60	0.48	0.38	0.36	0.34	0.32	0.55	0.23
Trypanosoma cruzi-negative 31	0.16	0.17	0.13	0.15	0.20	0.24	0.50	0.59
Trypanosoma cruzi-negative 32	0.52	0.41	0.35	0.34	0.34	0.33	0.57	0.23
Trypanosoma cruzi-negative 33	0.36	0.31	0.27	0.28	0.42	0.42	0.25	0.35
Trypanosoma cruzi-negative 34	0.40	0.36	0.30	0.30	0.28	0.27	0.52	0.57
Trypanosoma cruzi-negative 35	0.68	0.54	0.45	0.36	0.36	0.35	0.74	0.27
Trypanosoma cruzi-negative 36	0.70	0.30	0.33	0.25	0.23	0.20	0.45	0.47
Trypanosoma cruzi-negative 37	0.48	0.39	0.32	0.32	0.30	0.29	0.47	0.50
Trypanosoma cruzi-negative 38	0.52	0.40	0.36	0.40	0.38	0.36	0.63	0.21
Trypanosoma cruzi-negative 39	0.58	0.43	0.37	0.38	0.34	0.40	0.64	0.22
Trypanosoma cruzi-negative 40	0.43	0.34	0.28	0.32	0.29	0.26	0.45	0.20
Trypanosoma cruzi-negative 41	0.75	0.37	0.36	0.34	0.32	0.30	0.46	0.23
Trypanosoma cruzi-negative 42	0.49	0.46	0.29	0.41	0.34	0.28	0.49	0.23
Trypanosoma cruzi-negative 43	0.50	0.41	0.33	0.34	0.31	0.28	0.54	0.22
Trypanosoma cruzi-negative 44	0.40	0.30	0.27	0.28	0.26	0.24	0.37	0.19
Trypanosoma cruzi-negative 45	0.41	0.31	0.28	0.28	0.28	0.28	0.46	0.19

Trypanosoma cruzi-negative 46	0.27	0.23	0.22	0.23	0.22	0.21	0.33	0.12
Trypanosoma cruzi-positive 01	2.09	1.92	1.55	1.82	1.56	1.30	1.85	1.70
Trypanosoma cruzi-positive 02	0.79	0.70	0.51	0.55	0.48	0.41	0.87	0.33
Trypanosoma cruzi-positive 03	0.87	0.51	0.39	0.41	0.39	0.37	0.72	0.25
Trypanosoma cruzi-positive 04	1.85	1.72	1.35	1.43	1.22	1.01	1.67	1.06
Trypanosoma cruzi-positive 05	0.99	0.94	0.68	0.65	0.62	0.60	1.04	0.45
Trypanosoma cruzi-positive 06	1.55	1.37	1.17	1.18	1.04	0.90	1.51	0.87
Trypanosoma cruzi-positive 07	0.83	0.76	0.61	0.59	0.53	0.46	1.00	0.34
Trypanosoma cruzi-positive 08	1.54	1.34	1.14	0.11	0.46	0.80	1.48	0.82
Trypanosoma cruzi-positive 09	1.89	1.88	1.41	1.36	1.17	0.97	1.91	0.95
Trypanosoma cruzi-positive 10	1.20	0.95	0.83	0.88	0.78	0.69	1.07	0.73
Trypanosoma cruzi-positive 11	2.22	1.92	1.59	1.31	1.18	1.06	2.04	1.23
Trypanosoma cruzi-positive 12	1.04	1.06	0.81	0.67	0.66	0.65	1.01	0.66
Trypanosoma cruzi-positive 13	0.85	0.85	0.68	0.68	0.64	0.60	1.10	0.51
Trypanosoma cruzi-positive 14	1.09	1.03	0.78	0.71	0.67	0.62	1.10	0.48
Trypanosoma cruzi-positive 15	2.00	1.87	1.43	1.53	1.36	1.20	1.91	0.86
Trypanosoma cruzi-positive 16	1.34	1.26	1.03	1.08	0.61	0.13	1.32	0.53
Trypanosoma cruzi-positive 17	1.25	1.15	0.99	0.93	0.84	0.74	1.28	0.55
Trypanosoma cruzi-positive 18	0.82	0.56	0.51	0.61	0.49	0.36	0.64	0.30
Trypanosoma cruzi-positive 19	0.85	0.46	0.36	0.34	0.31	0.29	0.55	0.72
Trypanosoma cruzi-positive 20	0.85	0.58	0.53	0.48	0.45	0.42	0.69	0.27
Trypanosoma cruzi-positive 21	1.93	1.72	1.39	1.35	1.23	1.12	0.18	0.33
Trypanosoma cruzi-positive 22	2.46	2.33	1.88	1.95	1.78	1.60	2.32	1.07
Trypanosoma cruzi-positive 23	1.66	1.66	1.41	1.47	1.26	1.06	1.73	1.99
Trypanosoma cruzi-positive 24	2.66	2.46	2.00	2.06	1.80	1.54	2.44	1.26

Trypanosoma cruzi-positive 25	1.11	1.02	0.86	0.73	0.74	0.74	1.42	1.76
Trypanosoma cruzi-positive 26	1.91	1.69	1.41	1.55	1.34	1.13	1.67	1.36
Trypanosoma cruzi-positive 27	3.04	2.68	2.17	2.37	2.06	1.75	2.88	2.07
Trypanosoma cruzi-positive 28	1.03	0.89	0.79	0.58	0.53	0.48	0.89	0.37
Trypanosoma cruzi-positive 29	0.85	0.83	0.76	0.65	0.59	0.53	0.84	0.46
Trypanosoma cruzi-positive 30	0.87	0.43	0.33	0.34	0.32	0.29	0.58	0.21
Trypanosoma cruzi-positive 31	2.74	2.56	2.03	2.09	1.85	1.60	2.50	1.95
Trypanosoma cruzi-positive 32	0.84	0.44	0.38	0.42	0.37	0.32	0.63	0.30
Trypanosoma cruzi-positive 33	2.16	2.16	1.75	1.78	1.51	1.23	2.16	1.52
Trypanosoma cruzi-positive 34	2.33	2.05	1.92	1.63	1.45	1.27	1.95	1.58
Trypanosoma cruzi-positive 35	2.72	2.34	2.15	2.07	1.80	1.53	2.42	1.86
Trypanosoma cruzi-positive 36	0.84	0.40	0.29	0.27	0.24	0.21	0.55	0.22
Trypanosoma cruzi-positive 37	2.79	2.67	2.04	2.21	1.89	1.56	2.50	1.97
Trypanosoma cruzi-positive 38	1.71	1.83	1.22	1.23	1.13	1.02	1.46	1.03
Trypanosoma cruzi-positive 39	0.88	0.52	0.48	0.45	0.46	0.47	0.66	0.29
Trypanosoma cruzi-positive 40	1.11	1.05	0.90	1.31	1.13	0.95	1.08	1.28
Trypanosoma cruzi-positive 41	1.31	1.27	0.99	0.94	0.85	0.77	1.35	0.87
Trypanosoma cruzi-positive 42	2.32	2.35	1.86	1.96	1.69	1.42	2.30	1.82
Trypanosoma cruzi-positive 43	0.81	0.57	0.48	0.42	0.39	0.36	0.73	0.28
Trypanosoma cruzi-positive 44	2.18	1.95	1.56	1.43	1.32	1.20	2.04	1.24
Trypanosoma cruzi-positive 45	0.89	0.80	0.68	0.60	0.55	0.51	1.01	0.48
Trypanosoma cruzi-positive 46	0.84	0.86	0.65	0.58	0.51	0.44	0.87	0.38

Indirect ELISA – IBMP-8.3

Samples	IBMP-8.3							
	Time of exposure (DAYS)							
	0	52	104	156	206	260	312	364
*Trypanosoma cruz*i-negative 01	0.25	0.32	0.31	0.29	0.19	0.25	0.31	0.32
Trypanosoma cruzi-negative 02	0.50	0.48	0.45	0.41	0.31	0.66	0.24	0.41
Trypanosoma cruzi-negative 03	0.27	0.28	0.27	0.30	0.23	0.28	0.57	0.17
Trypanosoma cruzi-negative 04	0.43	0.36	0.36	0.43	0.32	0.36	0.35	0.32
Trypanosoma cruzi-negative 05	0.36	0.23	0.49	0.30	0.26	0.34	0.51	0.25
Trypanosoma cruzi-negative 06	0.50	0.30	0.49	0.40	0.31	0.38	0.57	0.23
Trypanosoma cruzi-negative 07	0.37	0.37	0.32	0.35	0.27	0.46	0.39	0.25
Trypanosoma cruzi-negative 08	0.34	0.31	0.35	0.34	0.28	0.50	0.29	0.21
Trypanosoma cruzi-negative 09	0.23	0.26	0.19	0.27	0.27	0.42	0.21	0.14
Trypanosoma cruzi-negative 10	0.39	0.34	0.33	0.37	0.33	0.60	0.20	0.19
Trypanosoma cruzi-negative 11	0.55	0.49	0.42	0.43	0.37	0.38	0.53	0.34
Trypanosoma cruzi-negative 12	0.52	0.50	0.61	0.51	0.41	0.49	0.27	0.31
Trypanosoma cruzi-negative 13	0.43	0.46	0.34	0.44	0.37	0.36	0.35	0.27
Trypanosoma cruzi-negative 14	0.29	0.25	0.24	0.29	0.29	0.26	0.09	0.23
Trypanosoma cruzi-negative 15	0.58	0.46	0.52	0.55	0.46	0.50	0.38	0.51
Trypanosoma cruzi-negative 16	0.35	0.40	0.35	0.36	0.35	0.42	0.36	0.22
Trypanosoma cruzi-negative 17	0.43	0.36	0.29	0.42	0.38	0.38	0.41	0.33
Trypanosoma cruzi-negative 18	0.42	0.39	0.35	0.37	0.37	0.53	0.29	0.45
Trypanosoma cruzi-negative 19	0.27	0.29	0.32	0.32	0.32	0.33	0.25	0.33
Trypanosoma cruzi-negative 20	0.31	0.26	0.27	0.35	0.36	0.33	0.28	0.20

Trypanosoma cruzi-negative 21	0.68	0.62	0.63	0.53	0.47	0.56	0.30	0.24
Trypanosoma cruzi-negative 22	0.52	0.57	0.40	0.46	0.40	0.43	0.45	0.27
Trypanosoma cruzi-negative 23	0.47	0.40	0.36	0.47	0.42	0.40	0.41	0.42
Trypanosoma cruzi-negative 24	0.30	0.31	0.26	0.29	0.32	0.25	0.21	0.38
Trypanosoma cruzi-negative 25	0.29	0.47	0.24	0.16	0.24	0.48	0.60	0.21
Trypanosoma cruzi-negative 26	0.49	0.39	0.30	0.31	0.35	0.26	0.21	0.40
Trypanosoma cruzi-negative 27	0.37	0.45	0.45	0.39	0.38	0.55	0.33	0.36
Trypanosoma cruzi-negative 28	0.33	0.35	0.29	0.30	0.35	0.55	0.42	0.22
Trypanosoma cruzi-negative 29	0.34	0.32	0.34	0.36	0.37	0.36	0.32	0.31
Trypanosoma cruzi-negative 30	0.43	0.41	0.38	0.44	0.45	0.41	0.36	0.25
Trypanosoma cruzi-negative 31	0.17	0.17	0.16	0.24	0.31	0.53	0.19	0.31
Trypanosoma cruzi-negative 32	0.50	0.38	0.43	0.42	0.41	0.38	0.37	0.28
Trypanosoma cruzi-negative 33	0.24	0.23	0.22	0.24	0.34	0.23	0.25	0.15
Trypanosoma cruzi-negative 34	0.66	0.58	0.58	0.51	0.53	0.50	0.28	0.36
Trypanosoma cruzi-negative 35	0.50	0.41	0.41	0.38	0.44	0.38	0.35	0.39
Trypanosoma cruzi-negative 36	0.42	0.77	0.37	0.30	0.39	0.36	0.42	0.26
Trypanosoma cruzi-negative 37	0.38	0.26	0.24	0.30	0.40	0.23	0.31	0.41
Trypanosoma cruzi-negative 38	0.49	0.38	0.40	0.45	0.51	0.43	0.25	0.29
Trypanosoma cruzi-negative 39	0.44	0.34	0.39	0.38	0.46	0.39	0.34	0.26
Trypanosoma cruzi-negative 40	0.41	0.37	0.33	0.37	0.47	0.33	0.51	0.57
Trypanosoma cruzi-negative 41	0.62	0.59	0.50	0.55	0.65	0.49	0.50	0.35
Trypanosoma cruzi-negative 42	0.38	0.36	0.34	0.36	0.49	0.40	0.28	0.24
Trypanosoma cruzi-negative 43	0.43	0.39	0.40	0.35	0.48	0.34	0.47	0.27
Trypanosoma cruzi-negative 44	0.33	0.31	0.27	0.29	0.54	0.33	0.28	0.31
Trypanosoma cruzi-negative 45	0.38	0.35	0.31	0.36	0.64	0.35	0.25	0.28

Trypanosoma cruzi-negative 46	0.23	0.25	0.21	0.28	0.64	0.27	0.36	0.46
Trypanosoma cruzi-positive 01	2.51	2.30	2.11	2.68	0.91	1.88	1.92	1.73
Trypanosoma cruzi-positive 02	1.42	1.28	1.23	1.29	0.38	0.92	0.98	0.90
Trypanosoma cruzi-positive 03	0.83	0.31	0.30	0.38	0.33	0.35	0.37	0.20
Trypanosoma cruzi-positive 04	2.62	2.08	1.93	2.44	1.16	1.74	2.08	1.76
Trypanosoma cruzi-positive 05	2.81	2.24	2.06	2.40	1.04	1.82	2.13	1.78
Trypanosoma cruzi-positive 06	2.06	1.97	1.82	2.04	0.91	1.46	1.34	1.48
Trypanosoma cruzi-positive 07	0.79	0.65	0.56	0.60	0.51	0.65	0.47	0.47
Trypanosoma cruzi-positive 08	2.16	1.88	1.75	1.98	1.19	1.46	1.35	1.50
Trypanosoma cruzi-positive 09	2.77	2.63	2.20	2.82	1.64	1.92	1.98	2.08
Trypanosoma cruzi-positive 10	1.23	1.06	1.10	1.00	0.73	0.91	0.67	0.73
Trypanosoma cruzi-positive 11	3.30	2.62	2.44	3.08	1.68	2.18	2.88	2.30
Trypanosoma cruzi-positive 12	0.85	0.39	0.38	0.41	0.67	0.42	0.32	0.28
Trypanosoma cruzi-positive 13	1.67	1.57	1.49	1.64	1.14	1.23	1.10	1.24
Trypanosoma cruzi-positive 14	1.54	1.35	1.35	1.32	1.11	1.00	0.98	1.06
Trypanosoma cruzi-positive 15	2.98	2.23	2.23	3.08	1.93	1.95	2.35	2.11
Trypanosoma cruzi-positive 16	2.02	1.95	1.85	2.23	1.66	0.17	1.77	1.38
Trypanosoma cruzi-positive 17	2.88	2.29	2.18	2.80	1.90	2.00	2.19	1.98
Trypanosoma cruzi-positive 18	1.72	1.62	1.60	1.65	1.63	1.26	1.48	1.24
Trypanosoma cruzi-positive 19	1.93	1.93	1.63	2.21	1.77	1.42	1.62	2.10
Trypanosoma cruzi-positive 20	1.18	0.93	0.93	0.81	1.11	0.75	0.66	1.44
Trypanosoma cruzi-positive 21	2.12	1.89	1.81	2.17	1.82	1.52	0.21	0.63
Trypanosoma cruzi-positive 22	2.10	1.93	1.70	2.00	1.74	1.43	1.09	1.50
Trypanosoma cruzi-positive 23	1.29	1.24	1.24	1.25	1.45	0.93	1.05	1.46
Trypanosoma cruzi-positive 24	1.91	1.66	1.69	1.89	1.75	1.28	1.61	0.78

Trypanosoma cruzi-positive 25	2.44	2.40	2.22	2.87	2.14	2.00	1.94	1.45
Trypanosoma cruzi-positive 26	1.96	1.91	1.81	2.11	1.87	1.44	1.27	1.32
Trypanosoma cruzi-positive 27	3.10	2.38	2.33	3.09	2.24	2.06	2.25	2.14
Trypanosoma cruzi-positive 28	0.81	0.52	0.54	0.48	1.06	0.43	0.31	0.35
Trypanosoma cruzi-positive 29	2.06	2.06	1.92	2.09	1.94	1.54	1.65	1.69
Trypanosoma cruzi-positive 30	1.77	1.53	1.46	1.62	1.87	1.18	1.54	1.17
Trypanosoma cruzi-positive 31	3.20	2.60	2.36	3.08	2.41	2.12	2.43	2.25
Trypanosoma cruzi-positive 32	1.31	1.34	1.26	1.21	1.88	0.87	0.90	0.89
Trypanosoma cruzi-positive 33	1.98	1.86	1.79	2.02	2.11	1.38	1.90	1.43
Trypanosoma cruzi-positive 34	2.49	2.30	2.31	2.55	2.35	1.73	2.10	1.80
Trypanosoma cruzi-positive 35	2.75	2.55	2.34	2.97	2.48	1.99	2.23	2.11
Trypanosoma cruzi-positive 36	0.85	0.34	0.32	0.31	1.46	0.29	0.27	0.25
Trypanosoma cruzi-positive 37	3.55	2.92	2.67	3.64	2.79	2.48	3.10	2.70
Trypanosoma cruzi-positive 38	2.99	2.71	2.48	3.11	2.54	2.19	1.66	2.07
Trypanosoma cruzi-positive 39	0.79	0.57	0.60	0.55	1.62	0.52	0.35	0.47
Trypanosoma cruzi-positive 40	2.41	2.22	2.07	2.64	2.54	1.76	1.87	1.93
Trypanosoma cruzi-positive 41	2.88	2.69	2.41	3.13	2.59	2.11	2.52	2.19
Trypanosoma cruzi-positive 42	2.13	2.10	1.83	2.04	2.38	1.50	1.94	1.65
Trypanosoma cruzi-positive 43	1.31	1.40	1.28	1.45	2.11	0.97	0.86	0.87
Trypanosoma cruzi-positive 44	2.19	1.78	1.75	2.35	2.52	1.51	1.60	1.59
Trypanosoma cruzi-positive 45	2.07	1.86	1.77	2.40	2.53	1.48	1.73	1.59
Trypanosoma cruzi-positive 46	2.85	2.16	2.17	2.86	2.80	1.87	2.38	2.04

Indirect ELISA – IBMP-8.4

Samples	IBMP-8.4							
	Time of exposure (DAYS)							
	**0**	**52**	**104**	**156**	**206**	**260**	**312**	**364**
*Trypanosoma cruz*i-negative 01	0.33	0.30	0.26	0.30	0.20	0.22	0.45	0.30
Trypanosoma cruzi-negative 02	0.79	0.37	0.34	0.37	0.25	0.35	0.40	0.34
Trypanosoma cruzi-negative 03	0.31	0.27	0.26	0.27	0.25	0.26	0.74	0.24
Trypanosoma cruzi-negative 04	0.37	0.32	0.35	0.32	0.31	0.33	0.37	0.36
Trypanosoma cruzi-negative 05	0.33	0.29	0.33	0.29	0.27	0.24	0.50	0.54
Trypanosoma cruzi-negative 06	0.46	0.43	0.34	0.43	0.30	0.36	0.60	0.32
Trypanosoma cruzi-negative 07	0.32	0.30	0.30	0.30	0.33	0.27	0.36	0.47
Trypanosoma cruzi-negative 08	0.34	0.31	0.33	0.31	0.29	0.34	0.55	0.56
Trypanosoma cruzi-negative 09	0.24	0.34	0.20	0.34	0.34	0.23	0.34	0.37
Trypanosoma cruzi-negative 10	0.38	0.39	0.32	0.39	0.31	0.32	0.51	0.57
Trypanosoma cruzi-negative 11	0.45	0.38	0.26	0.38	0.29	0.21	0.61	0.25
Trypanosoma cruzi-negative 12	0.68	0.42	0.34	0.42	0.29	0.36	0.70	0.31
Trypanosoma cruzi-negative 13	0.41	0.50	0.32	0.50	0.32	0.32	0.63	0.32
Trypanosoma cruzi-negative 14	0.28	0.25	0.20	0.25	0.30	0.23	0.19	0.47
Trypanosoma cruzi-negative 15	0.42	0.35	0.07	0.35	0.33	0.31	0.58	0.28
Trypanosoma cruzi-negative 16	0.55	0.34	0.28	0.34	0.39	0.31	0.25	0.47
Trypanosoma cruzi-negative 17	0.77	0.72	0.58	0.62	0.36	0.54	0.74	0.57
Trypanosoma cruzi-negative 18	0.36	0.31	0.31	0.31	0.31	0.29	0.46	0.30

Trypanosoma cruzi-negative 19	0.33	0.28	0.25	0.28	0.31	0.27	0.61	0.51
Trypanosoma cruzi-negative 20	0.43	0.31	0.30	0.31	0.30	0.28	0.36	0.30
Trypanosoma cruzi-negative 21	0.80	0.44	0.35	0.44	0.35	0.35	0.54	0.58
Trypanosoma cruzi-negative 22	0.39	0.41	0.47	0.41	0.30	0.30	0.55	0.34
Trypanosoma cruzi-negative 23	0.47	0.42	0.54	0.42	0.31	0.37	0.42	0.33
Trypanosoma cruzi-negative 24	0.30	0.31	0.26	0.31	0.43	0.24	0.27	0.21
Trypanosoma cruzi-negative 25	0.63	0.32	0.33	0.32	0.34	0.26	0.40	0.28
Trypanosoma cruzi-negative 26	0.25	0.27	0.22	0.27	0.35	0.21	0.30	0.41
Trypanosoma cruzi-negative 27	0.36	0.32	0.50	0.32	0.34	0.31	0.42	0.31
Trypanosoma cruzi-negative 28	0.29	0.30	0.30	0.30	0.26	0.26	0.30	0.23
Trypanosoma cruzi-negative 29	0.38	0.30	0.34	0.30	0.31	0.28	0.48	0.28
Trypanosoma cruzi-negative 30	0.46	0.41	0.37	0.41	0.28	0.35	0.51	0.40
Trypanosoma cruzi-negative 31	0.19	0.18	0.16	0.18	0.26	0.71	0.29	0.15
Trypanosoma cruzi-negative 32	0.47	0.38	0.34	0.38	0.34	0.31	0.50	0.32
Trypanosoma cruzi-negative 33	0.28	0.22	0.21	0.22	0.31	0.20	0.39	0.20
Trypanosoma cruzi-negative 34	0.76	0.86	0.66	0.56	0.37	0.59	0.80	0.78
Trypanosoma cruzi-negative 35	0.51	0.48	0.42	0.48	0.37	0.36	0.54	0.43
Trypanosoma cruzi-negative 36	0.42	0.35	0.44	0.35	0.33	0.27	0.53	0.30
Trypanosoma cruzi-negative 37	0.32	0.29	0.25	0.29	0.25	0.24	0.38	0.44
Trypanosoma cruzi-negative 38	0.53	0.43	0.35	0.43	0.49	0.34	0.54	0.30
Trypanosoma cruzi-negative 39	0.29	0.26	0.28	0.26	0.32	0.25	0.49	0.25
Trypanosoma cruzi-negative 40	0.31	0.35	0.29	0.35	0.48	0.27	0.52	0.34
Trypanosoma cruzi-negative 41	0.41	0.36	0.39	0.36	0.37	0.31	0.40	0.43
Trypanosoma cruzi-negative 42	0.38	0.34	0.28	0.34	0.28	0.29	0.49	0.56
Trypanosoma cruzi-negative 43	0.40	0.36	0.31	0.36	0.35	0.26	0.41	0.30

Trypanosoma cruzi-negative 44	0.38	0.35	0.36	0.35	0.37	0.28	0.38	0.27
Trypanosoma cruzi-negative 45	0.37	0.36	0.28	0.36	0.38	0.25	0.32	0.27
Trypanosoma cruzi-negative 46	0.25	0.27	0.23	0.27	0.54	0.21	0.43	0.48
Trypanosoma cruzi-positive 01	2.50	2.29	2.04	2.29	1.30	1.82	2.34	2.09
Trypanosoma cruzi-positive 02	0.87	0.79	0.69	0.79	0.56	0.63	0.70	0.67
Trypanosoma cruzi-positive 03	0.99	0.38	0.35	0.38	1.10	0.33	0.43	0.29
Trypanosoma cruzi-positive 04	2.51	2.59	2.07	2.59	1.85	1.95	2.69	2.20
Trypanosoma cruzi-positive 05	1.91	1.85	1.58	1.85	1.17	1.47	2.04	1.70
Trypanosoma cruzi-positive 06	2.34	2.15	1.80	2.15	1.52	1.69	1.74	1.75
Trypanosoma cruzi-positive 07	1.02	0.92	0.77	0.92	0.46	0.75	0.85	0.80
Trypanosoma cruzi-positive 08	2.54	2.17	1.82	2.17	1.63	1.69	2.09	1.83
Trypanosoma cruzi-positive 09	2.64	2.51	2.05	2.51	1.62	1.87	2.37	2.19
Trypanosoma cruzi-positive 10	1.34	1.14	0.96	1.14	1.37	0.86	1.18	0.92
Trypanosoma cruzi-positive 11	2.63	2.49	2.10	2.49	1.99	1.92	2.55	2.21
Trypanosoma cruzi-positive 12	2.24	2.20	1.86	2.20	1.72	1.68	2.21	1.83
Trypanosoma cruzi-positive 13	2.10	2.12	1.71	2.12	1.67	1.72	2.09	1.74
Trypanosoma cruzi-positive 14	2.46	2.57	1.95	2.57	2.27	2.01	2.45	2.07
Trypanosoma cruzi-positive 15	2.75	2.69	2.11	2.69	1.74	2.31	2.99	2.42
Trypanosoma cruzi-positive 16	2.23	2.20	1.85	2.20	2.23	0.16	2.24	1.91
Trypanosoma cruzi-positive 17	2.00	2.03	1.65	2.03	2.01	1.44	2.06	1.60
Trypanosoma cruzi-positive 18	1.81	1.71	1.58	1.71	2.01	1.32	1.84	1.48
Trypanosoma cruzi-positive 19	1.56	1.54	1.32	1.54	1.61	1.16	1.49	2.16
Trypanosoma cruzi-positive 20	1.45	1.45	1.22	1.45	1.69	1.13	1.56	1.39
Trypanosoma cruzi-positive 21	2.25	2.05	1.78	2.05	2.19	1.86	0.18	1.14
Trypanosoma cruzi-positive 22	2.42	2.44	1.92	2.44	1.74	1.82	1.98	1.90

Trypanosoma cruzi-positive 23	2.83	2.66	2.28	2.66	2.38	2.26	2.92	2.19
Trypanosoma cruzi-positive 24	2.86	2.86	2.33	2.86	2.54	2.39	3.00	2.40
Trypanosoma cruzi-positive 25	2.57	2.62	2.00	2.62	1.54	2.17	2.21	2.54
Trypanosoma cruzi-positive 26	2.74	2.61	2.15	2.61	2.42	2.10	2.02	2.19
Trypanosoma cruzi-positive 27	2.87	2.71	2.19	2.71	1.45	2.24	2.81	2.33
Trypanosoma cruzi-positive 28	1.51	1.39	1.22	1.39	1.61	1.00	1.27	1.21
Trypanosoma cruzi-positive 29	1.40	1.35	1.25	1.35	1.00	1.04	1.35	1.08
Trypanosoma cruzi-positive 30	1.56	1.69	1.30	1.69	2.30	1.00	1.33	1.20
Trypanosoma cruzi-positive 31	2.83	2.85	2.30	2.85	1.81	2.26	3.00	2.58
Trypanosoma cruzi-positive 32	0.87	0.86	0.75	0.86	1.62	0.57	0.77	0.66
Trypanosoma cruzi-positive 33	2.69	2.47	2.09	2.47	2.38	2.06	2.70	2.20
Trypanosoma cruzi-positive 34	2.98	2.70	2.32	2.70	2.47	2.26	2.73	2.51
Trypanosoma cruzi-positive 35	2.90	2.54	2.34	2.54	2.00	2.17	2.36	2.50
Trypanosoma cruzi-positive 36	0.84	0.50	0.41	0.50	0.95	0.28	0.62	0.40
Trypanosoma cruzi-positive 37	3.07	3.07	2.38	3.07	2.25	2.47	3.07	2.74
Trypanosoma cruzi-positive 38	2.15	2.39	1.72	2.39	1.59	1.61	1.77	1.62
Trypanosoma cruzi-positive 39	0.89	0.59	0.57	0.59	1.13	0.53	0.69	0.48
Trypanosoma cruzi-positive 40	1.58	1.54	1.23	1.54	1.70	1.31	1.66	1.51
Trypanosoma cruzi-positive 41	2.86	2.61	2.14	2.61	2.37	2.25	2.64	2.46
Trypanosoma cruzi-positive 42	3.03	2.72	2.31	2.72	2.15	2.23	3.19	2.51
Trypanosoma cruzi-positive 43	1.36	1.38	1.16	1.38	2.21	0.90	1.19	1.95
Trypanosoma cruzi-positive 44	2.37	2.31	1.87	2.31	2.12	1.89	2.49	2.10
Trypanosoma cruzi-positive 45	2.01	2.00	1.65	2.00	2.30	1.44	1.99	1.66
Trypanosoma cruzi-positive 46	2.19	2.25	1.79	2.25	2.47	1.43	2.36	1.72
